# Linear Polyethyleneimine-Based and Metal Organic Frameworks (DUT-67) Composite Hydrogels as Efficient Sorbents for the Removal of Methyl Orange, Copper Ions, and Penicillin V

**DOI:** 10.3390/gels9110909

**Published:** 2023-11-16

**Authors:** Luis M. Araque, Roberto Fernández de Luis, Arkaitz Fidalgo-Marijuan, Antonia Infantes-Molina, Enrique Rodríguez-Castellón, Claudio J. Pérez, Guillermo J. Copello, Juan M. Lázaro-Martínez

**Affiliations:** 1 Departamento de Ciencias Químicas, Facultad de Farmacia y Bioquímica, Universidad de Buenos Aires, Buenos Aires 1113, Argentina; lmaraque@conicet.gov.ar (L.M.A.); gcopello@ffyb.uba.ar (G.J.C.); 2 Consejo Nacional de Investigaciones Científicas y Técnicas (CONICET), Instituto de Química y Metabolismo del Fármaco (IQUIMEFA-UBA-CONICET), Buenos Aires 1113, Argentina; 3 BCMaterials, Basque Center for Materials, Applications and Nanostructures, UPV/EHU Science Park, 48940 Leioa, Spain; roberto.fernandez@bcmaterials.net (R.F.d.L.); arkaitz.fidalgo@ehu.eus (A.F.-M.); 4 Departamento de Química Orgánica e Inorgánica, Facultad de Ciencia y Tecnología, University of the Basque Country (UPV/EHU), 48940 Leioa, Spain; 5 Departamento de Química Inorgánica, Cristalografía y Mineralogía, Facultad de Ciencias, Universidad de Málaga, 29010 Malaga, Spain; ainfantes@uma.es (A.I.-M.); castellon@uma.es (E.R.-C.); 6 Consejo Nacional de Investigaciones Científicas y Técnicas (CONICET), Instituto de Investigaciones en Ciencia y Tecnología de Materiales (INTEMA), Facultad de Ingeniería, Universidad de Mar del Plata, Mar del Plata 7600, Argentina; cjperez@fi.mdp.edu.ar

**Keywords:** hydrogels, DUT-67, adsorption, multifunctional composites, emerging pollutants

## Abstract

This research explores the integration of DUT-67 metal organic frameworks into polyethyleneimine-based hydrogels to assemble a composite system with enough mechanical strength, pore structure and chemical affinity to work as a sorbent for water remediation. By varying the solvent-to-modulator ratio in a water-based synthesis path, the particle size of DUT-67 was successfully modulated from 1 μm to 200 nm. Once DUT-67 particles were integrated into the polymeric hydrogel, the composite hydrogel exhibited enhanced mechanical properties after the incorporation of the MOF filler. XPS, NMR, TGA, FTIR, and FT Raman studies confirmed the presence and interaction of the DUT-67 particles with the polymeric chains within the hydrogel network. Adsorption studies of methyl orange, copper(II) ions, and penicillin V on the composite hydrogel revealed a rapid adsorption kinetics and monolayer adsorption according to the Langmuir’s model. The composite hydrogel demonstrated higher adsorption capacities, as compared to the pristine hydrogel, showcasing a synergistic effect, with maximum adsorption capacities of 473 ± 21 mg L^−1^, 86 ± 6 mg L^−1^, and 127 ± 4 mg L^−1^, for methyl orange, copper(II) ions, and penicillin V, respectively. This study highlights the potential of MOF-based composite hydrogels as efficient adsorbents for environmental pollutants and pharmaceuticals.

## 1. Introduction

The quality and availability of water for human consumption are affected by industrial and urban activities, mainly due to the improper discharge of chemical and biological pollutants into water bodies. Particularly, dyes, heavy metal ions and emergent pollutants scape in different degrees from current wastewater treatment technologies, posing a risk to human health and the ecosystem equilibrium in general [1]. There is no regulation that restricts or determines a limit for the concentration of dyes in industrial effluents, as what is regulated is their use in products such as toys or textiles [2]. In the case of copper ions (Cu^2+^), the World Health Organization establishes a Cu^2+^ limit of 2 mg L^−1^ in drinking water [3,4]. Drugs such as penicillin V are considered emerging contaminants, and there is still not enough information on their effects on health or regulations for their disposal or presence in aquifers [4]. This worldwide issue is accentuated in isolated areas lacking adequate sanitation facilities. Therefore, it is imperative to develop materials and technologies to treat polluted water sources. One promising alternative is to create filter systems based on adsorbents. Adsorption is a low-cost and easy-to-implement technique that requires minimal energy. It has been widely and effectively used for the removal of pollutants from water [5]. Hydrogels, which production is quite affordable, are known to have high specific surface areas and adsorption capacities, as well as chemical versatility to modify their adsorption affinity. For these reasons, they have emerged as promising adsorbents [6]. Among them, polyethyleneimine-based gels have revealed outstanding capacities to retain different adsorbates, but most of them still exhibit poor mechanical resistance to deformation.

In a previous research work, our group developed a pH- and ionic strength-responsive hydrogel based on linear polyethyleneimine hydrochloride (L-PEI·HCl) [7]. Although the material had high swelling and adsorption capacities, it exhibited poor resistance to deformation, which hindered its manipulation, affecting its applicability. This hydrogel has a high affinity for transition metal ions, which allows us to evaluate the release of Cu^2+^ ions from montmorillonite materials with paramagnetic metal complexes [8].

The development of composite materials is a promising strategy to improve the mechanical properties of hydrogels. Composite materials feature an interface between the hydrogel polymer chains and the particulate filler that distributes the deformation applied to the material through this interface. The effect of the filler depends on three factors, i.e., particle size, the type of interaction between the filler and the polymeric matrix, and the filler dispersion and distribution degree along the matrix [9]. In this sense, Rao et al. developed a composite hydrogel by crosslinking gelatin with genipin, employing amino-functionalized microfibrillated cellulose as the filler [10]. The composite hydrogel exhibited a compressive strength of 1.52 MPa, which was 41.2 times higher than that of the hydrogel without the filler. In another study, a composite hydrogel was prepared by 3D printing gelatin methacrylate with nano-attapulgite as the filler, increasing the compressive strength and modulus 4.3 and 16.4 times, respectively [11]. Additionally, Chen et al. developed composite hydrogels based on polyvinyl alcohol with acidified carbon nanotubes as a filler [12]. By adding 2% of the filler, the Young’s modulus, maximum tensile stress, and toughness increased from 0.07 MPa, 108 kPa, and 83,819.2 J m^3^ to 0.185 MPa, 234 kPa, and 163,697.2 J m^3^, respectively [12,13,14].

Considering the potential of PEI hydrogels for water environmental remediation, the incorporation of sorbent-like metal organic framework (MOF) fillers into the PEI hydrogel matrix offers a dual advantage. This integration enhances both the mechanical properties and adsorption capacity of the hybridized system. MOFs are a class of porous materials that have gained popularity in recent years due to their application for water remediation purposes. Their very high specific surface areas, together with the chemically versatility to encode their frameworks and pore structures, has opened the way to their application for the removal and degradation via photocatalysis of myriad organic and inorganic pollutants [13,14,15,16]. The activity in this research area has been further intensified since the discovery of water-stable MOFs, including the DUT-67 one employed in this study as the filler of the PEI hydrogels. Further, the green synthesis protocols developed for trivalent and tetravalent metal carboxylate MOFs during the last years have facilitated the scale-up of these MOFs’ production with a high degree of control over their particle size. This is the case for the low-temperature water synthesis path applied to the crystallization of DUT-67 particles applied in this study [17,18,19]. As many other Zr-MOFs, the structure of DUT-67 is built up from the archetypal hexanuclear Zr_6_(OH)_4_O_4_(CO_2_^−^)_12_ clusters connected via eight 2,5-thiophenedicarboxilate linkers to form a micro- to mesoporous structure with two pores of different characteristics (i.e., diameter, pore window, and surface chemistry). All in all, the presence of thiophene fragments and uncoordinated positions occupied by modulator molecules within the zirconium cluster makes DUT-67 a versatile sorbent both for inorganics and organics with varied characteristics [20]. Few studies have assessed the use of fillers to enhance the mechanical properties of PEI-based hydrogels. There are no studies on the use of DUT-67 as a filler for PEI-based hydrogels or any other polymer. Furthermore, works on PEI-based hydrogels have predominantly focused on the use of the ramified architecture rather than on the linear one [17,21,22].

In this study, a high-yield low-temperature green synthesis of DUT-67 samples has been proposed. The modulation of DUT-67 crystallization by acetic acid has enabled a fine control over the particle size of the final material. Subsequently, DUT-67 nanoparticles were employed as a filler in the L-PEI·HCl hydrogels, which were obtained via a crosslinking with ethylene glycol diglycidyl ether (EGDE). The DUT-67 content in the composite was varied, and the physicochemical, thermal, and textural properties of the system were duly studied. The adsorptive properties of the composite gels for organic pollutants such as methyl orange (MO) azo dye, penicillin V (PEN) antibiotic, and copper ions (Cu^2+^) were investigated and correlated with the chemistry and porosity of the hydrogels before and after the incorporation of DUT-67 nanoparticles as an active filler in the system.

## 2. Results and Discussion

### 2.1. DUT-67 Synthesis and Characterization

**Figure 1:**
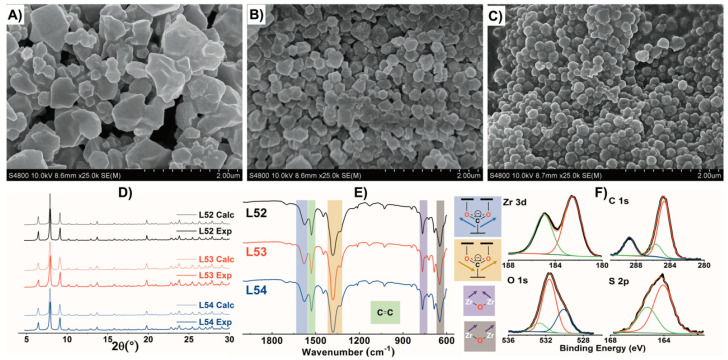
(**A**–**C**) SEM images, (**D**) PXRD patterns, (**E**) ATR—FTIR spectra of L52, L53, and L54; and (**F**) XPS spectra of L54.

The water-based green synthesis paths of MOFs have received important attention during the last years, including the ones reported for Zr-MOFs and, more specifically, for DUT-67. Considering the usual parameter spaces explored in these synthesis paths, we adapted the water-based crystallization of DUT-67 while modulating its particle size by controlling the concentration of the acetic acid modulator in the media [23]. As revealed by scanning electron microscopy (SEM) (Figure 1A–C), as the concentration of the modulator was lowered, the particle sizes of the DUT-67 samples (acetic acid) decreased from 1.01 ± 17 μm to 202 ± 24 nm. It is interesting to note that L54 exhibited a quite homogenous particle size shape in comparison to the ones obtained under higher concentrations of acetic acid [24,25].

The samples after the synthesis, washing, and activation process were characterized by powder X-ray diffraction (PXRD), attenuated total reflectance-Fourier-transform infrared spectroscopy (ATR-FTIR), FT Raman, and thermogravimetric analysis (TGA). First, the PXRD patterns for the three DUT samples were very similar to the calculated pattern obtained from the structural model reported by Drache et al. [26]. Second, a slight broadening of the diffraction maxima was observed as the particles size of the DUT-67 samples decreased (Figure 1D). The FTIR and Raman spectroscopies data (Figure 1E and S1) also confirmed that the acetic acid modulation did not cause significant changes in the chemistry of the DUT-67 samples. In detail, the FTIR spectra exhibited the characteristic absorption maxima ascribed to the bending vibrations of the Zr-O-Zr bonds (649 and 772 cm^−1^), the stretching vibrations of the O=C–O bonds (1574 (asymmetric) and 1393 (symmetric) cm^−1^), and the vibration of the C=C bond (1531 cm^−1^) [27]. The Raman spectra exhibited a characteristic peak at 1474 cm^−1^, which corresponded to the vibration of the C=C bond in the aromatic ring [28]. Although it is well known that the monocarboxylate modulators can induce significant linker defects on the crystal structure of Zr-MOFs, the thermogravimetry curves of the three DUT-67 samples were quite similar (Figure S2). Thus, there was expected to be a similar linker-defectiveness in the three of them. As expected, thermal degradation occurred in three steps, the first related to the dehydration occurring between 30 °C and 100 °C, the second ascribed to the acetic acid modulator release and the zirconium hexa-nuclear cluster dehydration from 100 to 350 °C, and the last one to the organic linker pyrolysis (350–550 °C).

High-pressure absorption isotherms acquired at 0 °C (Figure S3) showed 2 (L52), 2 (L53), and 5 mmol g^−1^ (L54) capacity to adsorb CO_2_ at 30 bar that were dependent on the modulator added to the synthesis. The lower the acetic acid concentration, the higher the capacity of the BET samples to capture CO_2_, and, in parallel, the higher the value of the surface area value obtained (L52: 841 m^2^ g^−1^, L53: 1083 m^2^ g^−1^, and L54: 1231 m^2^ g^−1^) after the fitting of the low-pressure range data. In view of the textural and morphological characteristics, L54 was selected as the sample for further characterization and integration into the polymeric PEI matrix. The surface chemistry of this sample was studied by means of X-ray photoelectron spectroscopy (XPS) (Figure 1F and Table S2). The Zr 3*d* core level spectrum showed the doublet ascribed to the spin orbit splitting of the Zr 3*d_5/2_* and 3*d_3/2_* levels located at 182.5 and 184.9 eV, respectively, in line with the values reported previously for DUT-67 [29]. For C 1*s*, three noticeable contributions were observed at 284.7, 285.6, and 288.8 eV, which corresponded to adventitious carbon layers containing aliphatic C-C and aromatic C=C bonds at 284.7 eV, C–OH and C-O-C at 285.6 eV, and carboxylate in the ligand at 288.8 eV. The O 1*s* core level spectrum was fitted with three contributions at 530.1, 531.6, and 532.7 eV, corresponding to the lattice oxygen atoms in the cluster, oxygen atoms of the ligand, and oxygen atoms from water molecules adsorbed into the material, respectively. Finally, the S 2*p* signal exhibited two contributions at 164.1 and 165.3 eV that were attributed to the S 2*p_3/2_* and 2*p_1/2_* doublet, respectively, of the S atoms in the thiophene ring [30,31].

**Figure 2:**
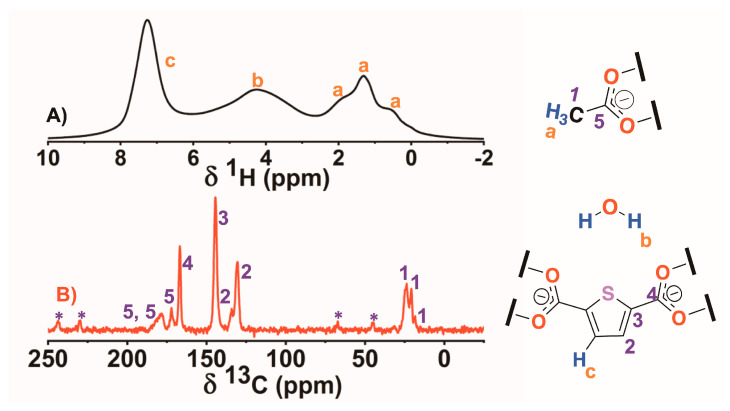
(**A**) ^1^H MAS (@32 kHz) and (**B**) ^13^C CP—MAS NMR (@15 kHz) spectra of DUT—67.

**Figure 3:**
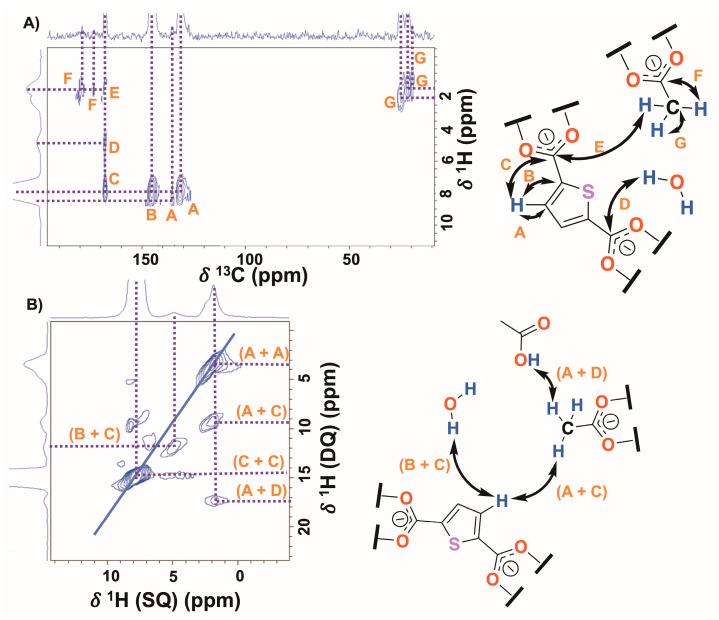
(**A**) The 2D ^1^H—^13^C HETCOR (@15 kHz), and (**B**) 2D ^1^H—^1^H SQ/DQ (@32 kHz) *ss*—NMR spectra of DUT—67.

*Solid-state* nuclear magnetic resonance spectroscopy (*ss*-NMR) experiments were also done to further investigate the bulk structure of the material. Figure 2A,B show the ^1^H magic angle spinning (MAS) and ^13^C cross-polarization and magic angle spinning (CP-MAS) NMR spectra of L54, respectively. In the ^1^H spectrum, signal c was assigned to the proton in the thiophene ring, while the other four signals did not belong to the DUT-67 components. Considering the shape and the chemical shift (~4.2 ppm), signal b was assigned to protons from free water molecules trapped within the MOF pores, even when the sample was oven-dried before the experiments. The three signals marked as a were assigned to methyl protons from three different acetic acid populations (modulator) that occupied the linker defective position of the zirconium hexanuclear clusters [32]. The signal widths confirmed, as expected, that the modulator populations are part of the structure and not molecules simply trapped within the material pores. The ^13^C *ss*-NMR spectrum exhibited the signals arising from two carbons (2 and 3) of the aromatic ring and an additional one belonging to the carboxylate groups (4) of the ligand. Two populations of linkers were found where carbon 2 had a distinct chemical arrangement and environment within the MOF structure. Additionally, the methyl carbon signals (1) revealed three populations of the acetic acid modulator. Moreover, two signals corresponding to the carboxylate carbons (5) were observed. One of the signals was very broad, suggesting the existence of at least two overlapping signals and, hence, and the presence of three acetate populations in the DUT-67structure. Finally, 2D *ss*-NMR experiments were performed to unravel the carbon hydrogen interactions within the DUT-67 framework. Figure 3 shows the 2D ^1^H-^13^C heteronuclear correlation (HETCOR) and ^1^H-^1^H single quantum/double quantum (SQ/DQ) results.

In the 2D ^1^H-^13^C HETCOR spectrum (Figure 3A), short- and long-range correlations were observed between the ligand hydrogen and the different carbons of the thiophene ring (A and B) and the carbon of the carboxylate groups (C), respectively. Interestingly, correlations were also observed between the protons of water molecules and the carbons of the carboxylate groups belonging to the ligand (D). Thus, these interactions would participate in the maintenance of the stability of the supramolecular structure. Furthermore, different chemical environments for the methyl (G correlations) and carboxylate (F correlations) groups of the modulator were evidenced through three different ^1^H and ^13^C populations. In addition, the correlation between the ligand and modulator (E) was clearly differentiated. In the 2D ^1^H-^1^H SQ/DQ spectrum (Figure 3B), correlations were observed between the ligand protons with free water molecules (B and C) and those of the modulator (A and C). Additionally, a correlation was detected between the modulator protons and an acidic hydrogen at ~12 ppm (A and D), which was not observed in the ^1^H-MAS experiments at 32 kHz. This correlation was assigned to carboxylic acid protons from modulator molecules found within the pores, as, in the structure, they are coordinated as carboxylates. These observations provide further evidence for the presence of free water molecules inside the pores, while the modulator is found both in the pores and as part of the MOF structure.

### 2.2. Composite Hydrogels Synthesis and Characterization

Composite hydrogels were synthesized via a click reaction in water between the amine groups of L-PEI·HCl and the epoxy ring of the EGDE component under mild conditions. Apart from the conditions implemented in the filler synthesis, the environmentally friendly synthesis conditions of click chemistry were adopted to minimize the ecological and economic impacts of the materials employed. The gelation consisted of a nucleophilic attack of the nitrogen from the polymer to the electrophilic carbon from a previously protonated epoxy ring of the crosslinker. Overall, a covalent bond between the two species was generated, giving rise to the reaction of both extremes of the EGDE molecules, the crosslinking of the different polymer chains, and finally, forming a 3D network structure. The same crosslinking reaction was effective when dispersed with the DUF-67 nanoparticles in the gelation media. Indeed, composites with 5, 10, and 15 wt% of the MOF with respect to the polymeric matrix were assembled by applying this strategy. In the last step, an acidic treatment was carried out to ensure the hydrolysis of any remaining epoxy rings to form 1,2-diol groups. The three composite hydrogels were named 5%D, 10%D, and 15%D.

**Figure 4:**
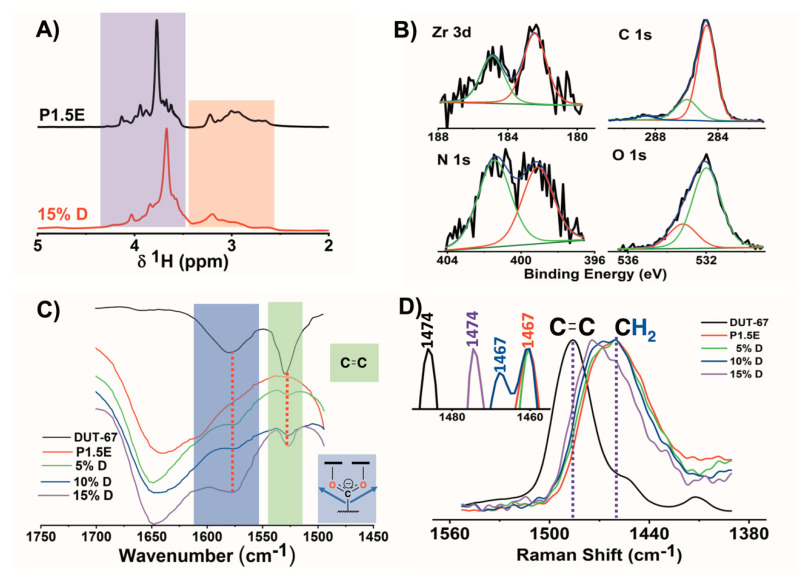
(**A**) ^1^H HRMAS NMR spectra of P1.5E and 15%D materials. (**B**) XPS spectra of 15%D. (**C**) ATR—FTIR and (**D**) FT Raman of DUT—67, pristine hydrogel, and composite hydrogels.

The influence of DUT-67 integration into the structure, chemistry, and mechanical properties of the composite hydrogels was characterized before their application for water remediation ends. First, the texture of the composite hydrogels hindered the detection of DUT-67 fingerprints by PXRD. Complementarily, the ^1^H high-resolution magic angle spinning (HRMAS) NMR data displayed two distinct zones with ^1^H chemical shifts ranging from 3.5 to 4.3 and 2.2 to 3.2 ppm, corresponding to the hydrogen atoms of the crosslinker and the polymer, respectively (Figure 4A). No resonance signals related to the filler were detected, which was expected due to the sample size and the low filler load (0.37 mg of the 2.5 mg employed to perform the experiment). Nonetheless, the resonance signals for the crosslinker and the polymer were affected by the MOF particles, which induced changes in the chemical environment and the structure of the hydrogel. These results also indicated that the distribution of the filler might be homogenous throughout the hydrogel structure.

The XPS spectra of 15%D corroborated the presence of zirconium ions with a similar Zr 3*d_5/2_* and 3*d_3/2_* binding energies (182.4 and 184.9 eV) compared to the ones of DUT-67 fillers (Figure 4B and Table S2). In the high-resolution C 1*s* spectrum, the three contributions observed at 284.7, 286.0, and 288.6 eV were ascribed to C–C and C=C bonds in the adventitious carbon and hydrogel chains (284.8 eV), to C–N and C–O bonds of the hydrogel chains (286.0 eV), and to carboxylate groups (288.6 eV). The contributions of C 1*s* in the composite hydrogels were consistent with those of P1.5E (Table S4). The contributions of carbon atoms from DUT-67 overlapped with those of the hydrogel components. A similar scenario was found for the XPS signals ascribed to O 1*s* (532.0 and 533.2 eV), which were almost identical to those of the pristine hydrogel (Table S4). Overall, the O 1*s* peak was ascribed to the sum of the contributions from the C–O bonds within the hydrogel structure and water molecules adsorbed on the material surface, respectively. Last, the bimodal N 1*s* spectrum (399.1 (C–N) and 401.5 (C–N^+^) eV) was related to neutral and protonated amine groups of the hydrogel [30,31].

ATR-FTIR and FT Raman spectroscopies can be used to confirm the presence of the inorganic and organic components of DUT-67 in composite hydrogels. If the MOF structure was altered during the hydrogel synthesis, the ligand would be in the form of carboxylic acid instead of carboxylate. Each form of the ligand absorbs IR radiation at different wavelengths. Figure S4 compares the FTIR spectra for DUT-67, pristine, and composite hydrogels. At first, there were no apparent differences among the spectra, which showed the characteristic signals for the hydrogel dominating the experimental contribution. These included the stretching vibration of the O–H and N–H bonds at 3000 cm^−1^, the vibration of the C–O–C (from the EGDE crosslinker) and C–N (from the polymer and the bond formed between the polymer and the crosslinker bridges) at 1070 cm^−1^, the bands corresponding to the vibrations of the aliphatic chains in both hydrogel components at 1454 and 2850 cm^−1^, and a low-intensity band at 1640 cm^−1^ from the bending vibration of the N–H bond of the secondary amines. However, after amplifying and normalizing the curves in the 1400–1700 cm^−1^ range (Figure 4C), the symmetric stretching vibration from the carboxylate (1574 cm^−1^) and the vibration of the C=C bonds from the aromatic ring (1531 cm^−1^) of the 2,5-thiophenedicarboxylate linker were detected [27]. Moreover, there was a direct correlation between the DUT-67content in the composite hydrogels and the intensity of the signals. Similarly, the normalization and amplification of the RAMAN spectra were necessary to extract the signature associated with DUT-67 from the ones of the polymeric hydrogel matrix (Figure S5). The characteristic vibrational modes of C–H (2800 and 3000 cm^−1^) and the bending vibration of -CH_2_- (1467 cm^−1^) dominated the Raman spectra. The characteristic vibration of the C=C bond from 2,5-thiophenedicarboxylate (1474 cm^−1^) was very close to the signals arising from the -CH_2_- group of the hydrogel [33].

**Figure 5:**
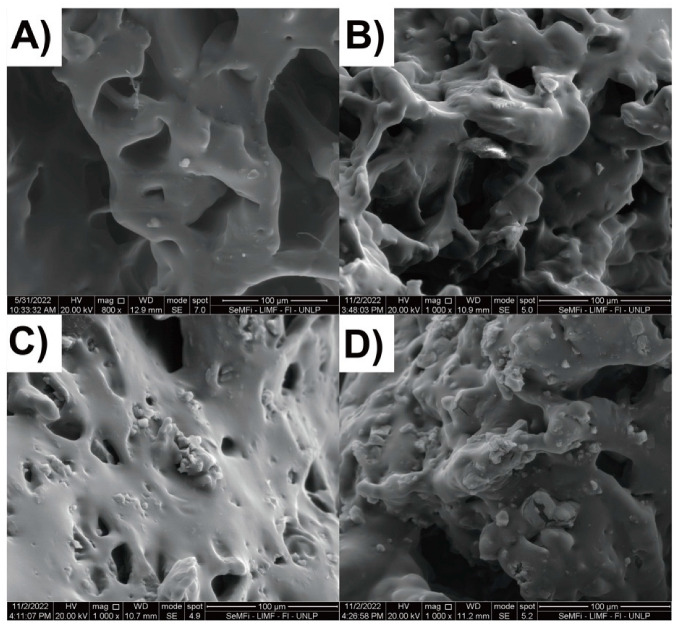
SEM images of (**A**) P1.5E, (**B**) 5%D, (**C**) 10%D, and (**D**) 15%D hydrogels.

To complete the structural characterization of the materials, the morphology of the samples was studied (Figure 5) by SEM, after being swollen in water and then lyophilized. Even though the structure of the gels collapsed after lyophilization, an interconnected porous structure could still be observed. The inclusion of DUT-67 particles did not affect the pore size of the hydrogels. Additionally, a partial agglomeration of filler particles was observed. Nevertheless, these agglomerated along with smaller DUT-67 particles and were distributed throughout the hydrogel matrix.

### 2.3. Thermal, Swelling, and Mechanical Properties of L-PEI·HCl@DUT-67 Hydrogels

The thermal behavior of the samples was studied through TGA (Figure S6). For all the samples a two-stage thermal evolution was observed. The first step occurred between 10 and 200 °C and accounted for the dehydration of the hydrogels, with a weight loss of approximately 8% of the initial mass. At temperatures above 230 °C, the pyrolysis of the hydrogel structure began, ending at approximately 570 °C. Overall, the weight loss ascribed to this second stage was approximately of 70% of the initial one. In agreement with the presence of DUT-67, the weigh percentage of the residue observed at 800 °C increased as the MOF loading did in the composite hydrogels, because the final thermal degradation product of DUT-67 was a ZrO_2_ residue. Although there were subtle differences on the TGA curve between the different hydrogels, we could not find a correlation between them and the degradation profile of DUT-67 sample.

**Figure 6:**
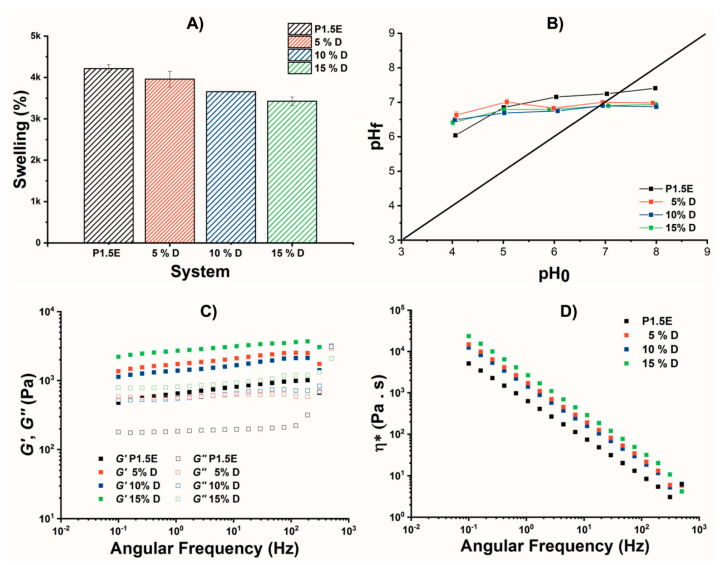
(**A**) Swelling capacities, (**B**) pH_pzc_, (**C**) *G*′ and *G*″, and (**D**) η* of P1.5E, 5%D, 10%D, and 15%D hydrogels.

Next, the swelling of the hydrogels was investigated, as illustrated in Figure 6A. The hydrogels rehydrated rapidly (~5 min), hindering the study of the kinetics. The swelling tended to decrease with the MOF loading. This tendency could be attributed to three factors. First, the filler particles restricted the freedom of the polymer chains to accommodate and support more water molecules in their network structure. Second, the interaction among polymer chains and the MOF particles (as indicated by the ^1^H HRMAS NMR experiments) reduced the number of available water adsorption sites for water adsorption into the hydrogel network. Third, the interactions between the two components leads to structural changes in the material, which affected its water retention capacity. Additionally, it is worth noting that DUT-67 is a micro-porous material that does not exhibit breathing-triggered morphological changes associated with water adsorption due to its rigid framework, and thus, it does not contribute to the overall swelling of the composites.

The point of zero charge (pH_pzc_), which is an important factor in evaluating the effect of pH in adsorption studies [34], was evaluated as well in our system, as shown in Figure 6B. It should be noted that the amine groups in the polymer were the most sensitive points to pH changes in the hydrogel. The p*K_a_* of PEI was approximately 10, indicating its basic nature [35]. During the hydrogel formation, the secondary amines were crosslinked and became tertiary, which were less basic, resulting in a decrease in the pH with a pH_PZC_ = 7.3 (pristine hydrogel). Moreover, DUT-67 softened the basicity of the material about 0.3 units, resulting in a pH_PZC_ = 7.0 (composite hydrogel), which was related to its interaction with the free electron pair of the amines of the hydrogel skeleton. Therefore, this result demonstrated that there was not only physical support of the DUT-67 into the hydrogel but a chemical interaction between the filler and the nitrogen atoms of the polymeric matrix.

The deformation response of the hydrogels studies in this work was studied by measuring several rheological parameters such as the storage (*G*′) and loss (*G*″) moduli and complex viscosity (η*). Figure 6C shows the *G*′ and *G*″ of the pristine hydrogel and the composite hydrogels. Within the studied frequency range (0.1–500 Hz), all four materials exhibited a linear viscoelastic response. For the four hydrogels, *G*′ (which was related to the elastic response) was higher than *G*″ (which was related to the viscous response). This behavior indicated that the three-dimensional network of the hydrogels presented a solid-like and elastic behavior [12]. On the other hand, the *G*′ and *G*″ values increased concomitantly with the percentage of DUT-67, pointing out that MOF-particles increased the material resistance to the deformation of the hydrogel. This phenomenon could be predicted, since the results of ^1^H HRMAS and pH_pzc_ allowed inferring that the polymer chains and the filler interacted with each other. Additionally, SEM images revealed that DUT-67 particles were distributed throughout the matrix. The complex viscosity (η*) is related to the stability of the hydrogel structure [36]. Figure 6D shows η* values as a function of the angular frequency for the pristine hydrogel and the composite hydrogels. For all materials, η* decreased linearly as the frequency increased. This finding indicates that the materials behaved as non-Newtonian fluids with a pseudo-plastic character [37,38]. The 15%D hydrogel presented the highest η* values, which decreased and as the filler content decreased, with the pristine hydrogel presenting the lowest values. This finding further supports the hypothesis that DUT-67 is acting as a mechanical reinforcement of the three-dimensional network of the hydrogels obtained, since it interacts adequately with the polymer chains and is distributed throughout the entire matrix, allowing for a greater stress-bearing capacity of the materials.

### 2.4. Functional Assessment of PEI@DUT-67 Hydrogels as Sorbents for Water Remediation

Due to its mechanical and chemical properties, 15%D was selected to assess its capacity and kinetics for the adsorption of MO, PEN antibiotic, and Cu^2+^ metal ions. To this end, experimental adsorption kinetics and isotherms data were obtained, and these data were fitted to mathematical models described in Experimental Section S1. Since the fittings were performed with non-linearized data, chi-square (*χ*^2^) and the residual sum of squares (*RSS*) were taken as the criteria to evaluate their degree of matching. The kinetics models used to fit the data were the pseudo-first order, pseudo-second order, and the Freundlich modified models. The isotherm models used to fit the data were the Langmuir and Freundlich models.

**Figure 7:**
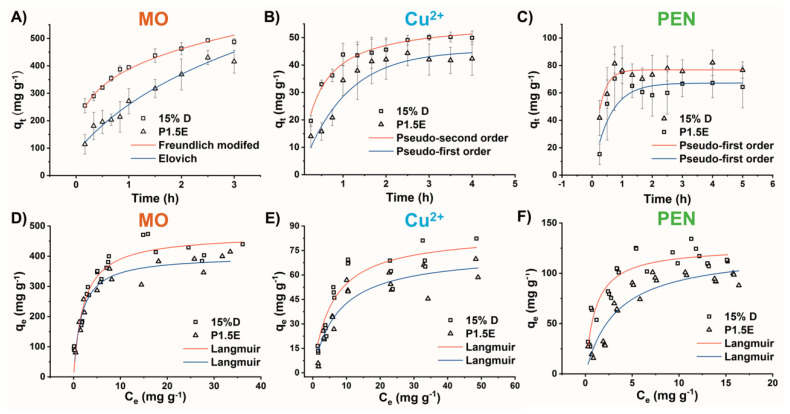
Adsorption kinetics of (**A**) MO, (**B**) Cu^2+^, and (**C**) PEN by the hydrogels and kinetics models fittings. Adsorption isotherms of (**D**) MO, (**E**) Cu^2+^, and (**F**) PEN and fittings to the Langmuir model.

The experimental adsorption rates of MO at pH = 6, along with the modeled kinetic data, are presented in Figure 7A and Table S5, respectively. The adsorption process for both hydrogels exhibited rapid kinetics, reaching equilibrium after approximately 120 min of contact. The experimental data were better fitted by the Freundlich modified model for the 15%D hydrogel, while the Elovich model provided a better fit for P1.5E. In the case of P1.5E, the suitability of the Elovich model suggested the presence of heterogeneous adsorption sites with chemisorption as the rate-controlling step of the process [39]. Since MO is an anionic pollutant and the hydrogel has a positive charge density, electrostatic attraction likely played a role in the adsorption process [40]. Conversely, for the 15%D hydrogel, the kinetics of MO adsorption was not controlled by the intra-particle diffusion process, as indicated by the value of *m* > 2. In general, adding DUT-67 particles altered the response of the material improving its kinetics of adsorption but also the adsorption capacity for a fixed MO concentration.

The experimental and modeled adsorption kinetics of P1.5E and 15%D for the capture of Cu^2+^ at pH = 4 are presented in Figure 7B and Table S6, respectively. Like the adsorption of MO, the adsorption of Cu^2+^ was rapid, even faster than that of MO. In this case, equilibrium was achieved after approximately 60 min. The interaction between Cu^2+^ ions and nitrogen atoms from secondary amines became stronger as stable coordinated complexes were formed. The experimental data were better fitted by the pseudo-second order model for the 15%D hydrogel, and by the pseudo-first model for P1.5E. However, for both hydrogels, the *χ*^2^ and *RSS* values (Table S6) of the pseudo-first and pseudo-second order models were found to be similar. Thus, the adsorption kinetics of Cu^2+^ by both hydrogels could be adequately described by both models, indicating that the adsorption of Cu^2+^ involved physical and chemical adsorption processes [41,42,43,44,45].

The adsorption rates of PEN at pH = 4 and the modeled kinetic data are presented in Figure 7C and Table S7, respectively. The adsorption of PEN was the most rapid of the three pollutants studied, reaching equilibrium after approximately 45 min. At pH = 4, the hydrogel amine groups were protonated (positively charged), while most carboxylic acid molecules were deprotonated (negatively charged), suggesting an electrostatic interaction between the material surface and the PEN molecules. For both P1.5E and the 15%D hydrogel, the experimental data were better fitted by the pseudo-first model. However, the values of the evaluation criteria for the pseudo-first and pseudo-second order models exhibited only a slight difference, suggesting that the controlling step of PEN adsorption involved physical and chemical processes.

Adsorption isotherms describe the medium conditions under which the equilibrium is reached between the adsorbate, that interacts with the host, and the dissolved species in the liquid phase, at a constant temperature. By fitting the experimental data obtained from the adsorption isotherm experiments to mathematical models, the mechanism of the adsorption and the maximum capacity (*q_e_*) to host the adsorbate by the adsorbent can be determined. That is, it delivers information about the number of adsorption sites and the strength/affinity of the interaction between the host and the guest. In this study, the adsorption isotherms of MO, Cu^2+^, and PEN on the hydrogels at different initial concentrations and at 25 °C were determined. The fitted parameters are shown in Figure 7D–F, and Tables S8, S9 and S10, respectively. For all three pollutants (MO, Cu^2+^, and PEN), the experimental isotherms data for P1.5E and the 15%D hydrogel were better fitted by the Langmuir model, indicating that MO, PEN, and Cu^2+^ could form a monolayer on the hydrogel surface, favoring the chemical adsorption process.

The experimental values of the maximum adsorption capacities (*q_max_*) for MO were 381 ± 24 mg g^−1^ and 420 ± 14 mg g^−1^, for P1.5E and 15%D, respectively, while the calculated values were 402 ± 14 mg g^−1^ and 473 ± 21 mg g^−1^. For Cu^2+^, the experimental *q_max_* values were 59 ± 9 mg g^−1^ and 71 ± 9 mg g^−1^, with calculated values of 74 ± 6 mg g^−1^ and 86 ± 6 mg g^−1^, for P1.5E and 15%D, respectively. Finally, the experimental *q_max_* values for PEN were 98 ± 9 mg g^−1^ and 115 ± 4 mg g^−1^, with calculated values of 123 ± 9 mg g^−1^ and 127 ± 4 mg g^−1^, for P1.5E and 15%D, respectively. All experimental and calculated values exhibited differences within a range of 4–11%, which is considered acceptable. Additionally, the synergistic effect between both components of the composite hydrogel was evident, as the adsorption capacity for 15%D was consistently higher than that of P1.5E for the three pollutants. Complementarily, the adsorption of pollutants on the composite hydrogels was studied by using ATR-FTIR spectroscopy (Figures S7–S9). The adsorption process was evident for MO, where absorption bands were added to those of hydrogels due to the high content in relation to the adsorbent. However, no significant changes were shown for Cu^2+^ ions and PEN due to the lower uptake by the adsorbents compared to MO. In general, it is difficult to visualize the adsorption process with this technique, because the matrix masks the absorption signals of the adsorbed molecules.

Considering the results from the functional assessment of our hydrogels, the adsorption kinetics and capacity of composite 15%D were always faster and higher than the observed ones for P1.5E, indicating a synergistic effect between both porous components, the hydrogel network structure and the micropore space of the DUT-67 nanoparticles. Similar synergic effects have been reported for polymer/MOF composite systems, where the kinetics and capacity of the systems overcame the average of their separate components [46,47].

### 2.5. Effect of the pH on the Adsorption Capacity

The pH value plays a crucial role in the adsorption process as it can influence the speciation and the charge of both the adsorbent and the adsorbate. To investigate the effect of the pH on the adsorption capacities of P1.5E and 15%D, adsorption experiments with MO and PEN at pH = 4, 7, and 10 were conducted, and the results are presented in Table 1. For Cu^2+^ ions, the effect of the pH on the adsorption process could not be studied, as Cu^2+^ precipitates as copper hydroxide at pH ≥ 5.2 [48]. Notably, for both materials, as the pH values increased, a decrease in the adsorption capacity was observed. This outcome was anticipated due to the nature of the hydrogels and pollutants involved. At pH ≥ 4, most of the MO (p*K_a_* = 4.3) and PEN (p*K_a_* = 4.3) molecules were negatively charged. Consequently, at pH = 4, the secondary and tertiary amine groups on the polymer were protonated, enabling the material to interact effectively with the negatively charged MO and PEN. As the pH increased, the amine groups in the polymer became deprotonated, leading to a reduced capacity for the hydrogels to interact electrostatically with the pollutant molecules [7].

### 2.6. Competitive Adsorption

Competitive adsorption experiments on a binary mixture were conducted to assess the effect of multiple pollutants on the adsorption process. The binary mixture consisted of Cu^2+^ and PEN, as each interacted differently with the materials. The experiments employed the same initial molar concentration, and the results are presented in Table 2.

**Figure 8:**
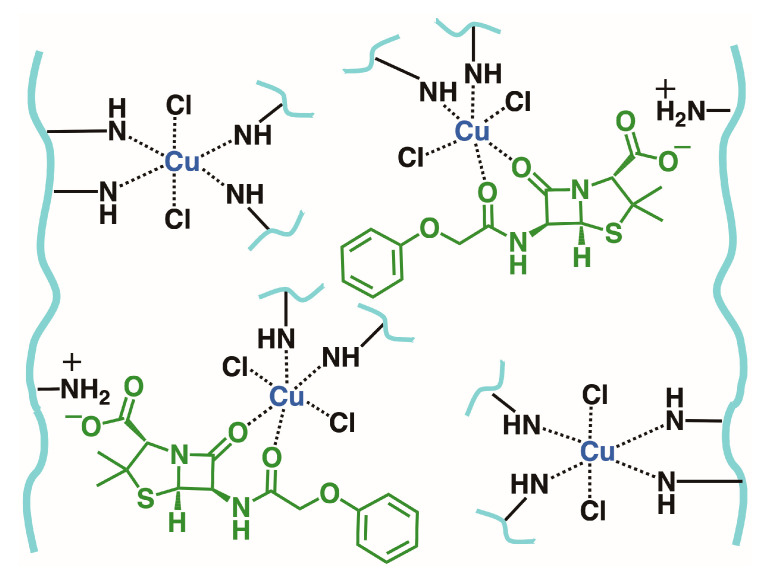
Diagram of the interaction between Cu^2+^ and PEN within the material. The secondary amine groups are simplified as —NH for clarity.

Once again, the synergistic effect between both components in 15%D was observed, with its adsorption capacity being higher than that of P1.5E in all cases. In the case of the mixture, both P1.5E and 15%D showed a preference for Cu^2+^ ions, resulting in an increase in the Cu^2+^ adsorption capacity, while decreasing the PEN adsorption capacity. The opposite was expected, since the association (*k_a_*) constant from the Langmuir model was higher for PEN than for Cu^2+^ (Tables S9 and S10, respectively). This result can be attributed to the chemical nature of PEN: a β-lactam compound prompt to coordinate metal ions [49,50]. After being adsorbed, PEN molecules can coordinate Cu^2+^ ions with the nitrogen atoms of the PEI structure, as illustrated in Figure 8, thereby increasing the capacity of the material to uptake Cu^2+^ from the solution.

## 3. Conclusions

The modulation of the particle size of DUT-67 was achieved in a green-synthesis path by controlling the water-to-modulator ratios. Through experiments using *ss*-NMR spectroscopy, the presence of (i) modulator molecules likely coordinated to the zirconium hexanuclear clusters of the DUT-67 structure and (ii) water molecules adsorbed into the pores were identified. XPS, NMR, FTIR, and FT Raman studies confirmed the presence and interaction of the DUT-67 particles with the polymeric chains within the hydrogel network. The composite hydrogel with 15% filler content exhibited the highest resistance to deformation, indicating a reinforcement of the hydrogel network structure by the porous MOF filler. Additionally, 15%D showed an improved adsorption capacity and kinetics as compared to the pristine hydrogel. The adsorption of MO, Cu^2+^ ions, and PEN on the hydrogels involved both physical and chemical adsorption processes. The Langmuir model provided the best fit for the adsorption isotherms, suggesting a monolayer-type adsorption of the pollutants on the hydrogel surface. The adsorption capacity decreased as the pH increased, as the deprotonation of amine groups in the polymer reduced the interaction with the negatively charged pollutants. In the binary mixture experiments, the hydrogels showed a preference for Cu^2+^ ions over PEN, resulting in an increased Cu^2+^ adsorption capacity and a decreased PEN adsorption capacity.

## 4. Materials and Methods

### 4.1. Materials

L-PEI·HCl (~87 kDa) was synthesized as described previously [51]. Analytical grade EGDE (TCI—Portland, OR, USA), and 2,5-thiophenedicarboxylic acid (H_2_TDC), acetic acid, zirconium dichloride oxide (ZrOCl_2_·8H_2_O), sodium acetate, hydrochloric acid, sodium hydroxide, MO, PEN, phosphate buffer, sodium chloride, copper(II) sulphate, and D_2_O (Merck—Rahway, NJ, USA) were of analytic grade and were used without further purification.

### 4.2. DUT-67 Synthesis

The DUT-67 samples were prepared by a high-yield water-based reflux-synthesis described previously by Reinsch et al. [52]. with some modifications. Briefly, 6.44 g (20 mmol) of ZrOCl_2_·8H_2_O were dissolved in 100 mL of water and acetic acid solution, and then, 2.29 g (13.3 mmol) of H_2_TDC were added to the reactor under stirring conditions. The concentration of acetic acid in the water solution was employed as the tool to control the particle size of the final DUT-67 material, as detailed in Table S1. The mixture was then heated at 95 °C and kept under vigorous stirring for 1 h. It is important to note that a white precipitate was generated after the addition of the organic linker to the ZrOCl_2_·8H_2_O solution. The DUT-67 particles were recovered by centrifugation (19,000 rpm, 30 min), redispersed twice in aqueous sodium acetate solution (15 mL, 0.1 M) and water (15 mL), and finally dried at 100 °C overnight. After drying, the solids were milled and named L52 (3.70 g), L53 (3.95 g), and L54 (3.87 g).

### 4.3. MOF@L-PEI·HCl Composite Hydrogels Synthesis

The L54 sample was employed as the filler to assemble the MOF@L-PEI·HCl composite hydrogels. First, DUT-67 200 nm particles were dispersed in 6.4 mL of H_2_O and sonicated for 1 h. The concentration of the MOF particles in the dispersion was controlled to obtain composite hydrogels with 5, 10, and 15 wt_%_ of the filler that were added to 6.4 mL of H_2_O. The mixture was sonicated for 1 h. Then, 320 mg (~1.7 mmol of N) of L-PEI·HCl and 0.8 mL (2.6 mmol) of EGDE were added to the MOF-dispersions. The solutions were heated at 90 °C for 180 min while stirring. After the synthesis, the hydrogels were swelled with distilled water, and further washes were performed to remove the unreacted species. The samples were sieved through a 1000 µm mesh to homogenize the particle size. Residual epoxide groups were hydrolyzed with 6 M HCl for 1 h. Finally, the samples were washed three times with 10 mM phosphate buffer to equilibrate to pH = 6 and then with distilled water. The initial L-PEI·HCl hydrogel and the composite ones obtained after the integration of the DUT-67 particles were named P1.5E, 5%D, 10%D, and 15%D, respectively. The reaction yields were calculated considering the initial mass of both precursors and the filler.

### 4.4. Characterization Techniques

Details related to the characterization techniques can be found in the Supplementary Materials (Experimental S1).

The morphology and hydrogel cross-section microstructure of the DUT-67 samples were studied by SEM. PXRD patterns of the DUT-67 samples were obtained using a Panalytical X ´pert CuKα diffractometer. ATR-FTIR and FT Raman spectra were recorded on a Nicolet iS50 spectrometer (Thermo Scientific—Waltham, MA, USA) using a one-reflection diamond crystal. HRMAS NMR and *ss*-NMR spectra were acquired with a Bruker Avance-III HD spectrometer equipped with a 14.1 T narrow bore magnet. The TGA was recorded with a TGA-50 Shimadzu. XPS analysis was carried out with a Physical Electronics (Versa-Pro II) operating with a monochromatic X-ray source Al (k-alpha) of photons at 1486 eV under ultra-high vacuum using a pressure of 10^−6^ Pa. CO_2_ high-pressure adsorption isotherms were acquired using ISorb-1 equipment from 0 to 30 bars and after activating the sample at 120 °C for 4 h. The BET surface areas were calculated following the protocol described by Kim et al. [53] and considering the fitting of the data between 0.05 and 0.35 bars. The viscoelastic behavior was determined with an Anton Paar rotational rheometer (MCR-301).

### 4.5. Kinetic and Adsorption Capacity Assessment

Adsorption tests were conducted in batches at room temperature (25 °C) and with constant stirring (100 rpm). Adsorption isotherms data were determined using 5 mg of dried hydrogel added to an aqueous solution (100 mL) of MO, Cu^2+^, or PEN with concentrations ranging from 4 to 45 mg L^−1^, 6 to 60 mg L^−1^, and 2 to 18 mg L^−1^, respectively. The concentration difference after reaching the equilibrium allowed for estimating the adsorption capacity of each of the studied materials at a given concentration of the pollutant. In parallel, the kinetics of the adsorption were determined by measuring the sorbate concentration decay in the solution supernatant during the adsorption process. To this end, MO, Cu^2+^, and PEN solutions with 60, 30, and 12 mg L^−1^ concentrations were employed to perform the experiments.

The concentrations of MO, Cu^2+^, and PEN before, during, and after adsorption were determined by UV Vis spectroscopy. MO presented a characteristic peak at 468 nm. For Cu^2+^, the chromogenic complexing agent 1-(2-pyridylazo)-2-naphtol (PAN) [54] was used, obtaining a Cu^2+^ complex that presented a characteristic absorption peak at 548 nm. PEN presented a characteristic peak at 220 nm. For the three target pollutants studied in this work, calibration curves were constructed. The experimental data obtained from the adsorption experiments were adjusted to kinetics and isotherm models (Experimental S1).

## Figures and Tables

**Table 1:** Effect of the pH on the adsorption capacities of MO and PEN by P1.5E and 15%D hydrogels.

	pH 4		pH 7		pH 10	
System	MO (mg g^−1^)	PEN (mg g^−1^)	MO (mg g^−1^)	PEN (mg g^−1^)	MO (mg g^−1^)	PEN (mg g^−1^)
P1.5E	696 ± 4	98 ± 9	462 ± 9	24 ± 3	78 ± 5	1.6 ± 1.0
15%D	799 ± 32	115 ± 4	584 ± 20	29 ± 3	92 ± 11	2.3 ± 0.6

**Table 2:** Competitive adsorption of Cu^2+^ and PEN by the hydrogels.

System	Cu^2+^ (mmol g^−1^)	PEN (mmol g^−1^)	Cu^2+^ Mixed (mmol g^−1^)	PEN Mixed (mmol g^−1^)
P1.5E	0.20 ± 0.01	0.32 ± 0.02	0.34 ± 0.06	0.22 ± 0.01
15%D	0.38 ± 0.02	0.36 ± 0.01	0.59 ± 0.02	0.29 ± 0.01

## Data Availability

The data presented in this study are available in the Supplementary Material.

## Supplementary Materials

The following supporting information can be downloaded at: https://www.mdpi.com/article/10.3390/gels9110909/s1, Experimental S1. Characterization techniques. Experimental S2. Kinetic and Isotherm models. Figure S1. FT Raman spectra f L52, L53, and L54; Figure S2. TGA curves of L52 and L53; Figure S3. High pressure CO_2_ adsorption isotherms at 0 °C of L52, L53, and L54; Figure S4. ATR-FTIR spectra of L54, P1.5E, 5%D, 10%D, and 15%D; Figure S5. FT RAMAN spectra of L54, P1.5E, 5%D, 10%D, and 15%D; Figure S6. TGA curves of L54, P1.5E, 5%D, 10%D, and 15%D; Figure S7. ATR-FTIR spectra of 15% D after MO adsorption; Figure S8. ATR-FTIR spectra of 15% D after Cu^2+^ adsorption; Figure S9. ATR-FTIR spectra of 15% D after PEN adsorption. Table S1. Solvent (water): modulator (acetic acid) proportions for DUT-67 synthesis; Table S2. Fitting parameters from XPS data of L54; Table S3. Fitting parameters from XPS data of 15%D; Table S4. Fitting parameters from XPS data of P1.5E; Table S5. Kinetic parameters for MO adsorption; Table S6. Kinetic parameters for Cu^2+^ adsorption; Table S7. Kinetic parameters for PEN adsorption; Table S8. Isotherm model parameters for MO adsorption; Table S9. Isotherm model parameters for Cu^2+^ adsorption; Table S10. Isotherm model parameters for PEN adsorption. References [55,56,57,58,59,60,61,62,63,64,65,66].
