# Supplementary material for: Linear Polyethyleneimine-Based and Metal Organic Frameworks (DUT-67) Composite Hydrogels as Efficient Sorbents for the Removal of Methyl Orange, Copper Ions, and Penicillin V
Source: Gels. 2023 Nov 16;9(11):909. doi: 10.3390/gels9110909 (PMC10671452; doi:10.3390/gels9110909)
Supplement: Supplementary file 1 [file gels-09-00909-s001.zip › gels-2713504-supplementary.pdf]

‘Supplementary Material’

Linear Polyethyleneimine-Based and Metal Organic Frameworks (DUT-67) Composite Hydrogels as Efficient Sorbents for the Removal of Methyl Orange, Copper Ions, and Penicillin V

Luis M. Araque [1,2], Roberto Fernández de Luis [3], Arkaitz Fidalgo-Marijuan [3,4], Antonia Infantes-Molina [5], Enrique Rodríguez-Castellón [5], Claudio J. Pérez [6], Guillermo J. Copello [1,2] and Juan M. Lázaro-Martínez [1,2,*]

[1]Departamento de Ciencias Químicas, Facultad de Farmacia y Bioquímica, Universidad de Buenos Aires, 1113, Buenos Aires, Argentina; lmaraque@conicet.gov.ar (L.M.A.); gcopello@ffyb.uba.ar (G.J.C.)
[2]Consejo Nacional de Investigaciones Científicas y Técnicas (CONICET), Instituto de Química y Metabolismo del Fármaco (IQUIMEFA-UBA-CONICET), 1113, Buenos Aires, Argentina
[3]BCMaterials, Basque Center for Materials, Applications and Nanostructures, UPV/EHU Science Park, 48940, Leioa, Spain; roberto.fernandez@bcmaterials.net (R.F.d.L.); arkaitz.fidalgo@ehu.eus (A.F.-M.)
[4]Departamento de Química Orgánica e Inorgánica, Facultad de Ciencia y Tecnología, University of the Basque Country (UPV/EHU), 48940, Leioa, Spain
[5]Departamento de Química Inorgánica, Cristalografía y Mineralogía, Facultad de Ciencias, Universidad de Málaga, 29010, Malaga, Spain; ainfantes@uma.es (A.I.-M.); castellon@uma.es (E.R.-C.)
[6]Consejo Nacional de Investigaciones Científicas y Técnicas (CONICET), Instituto de Investigaciones en Ciencia y Tecnología de Materiales (INTEMA), Facultad de Ingeniería, Universidad de Mar del Plata, 10850, Mar del Plata, Argentina; cjperez@fi.mdp.edu.ar
*Correspondence: lazarojm@ffyb.uba.ar; Tel.: +54-11-5287-4323

Table of Contents:

Content	*Page*
Experimental S1	S3
Experimental S2	S6
Figure S1	S8
Figure S2	S8
Figure S3	S9
Figure S4	S9
Figure S5	S10
Figure S6	S10
Figure S7	S11
Figure S8	S11
Figure S9	S12
Table S1	S13
Table S2	S14
Table S3	S15
Table S4	S16
Table S5	S17
Table S6	S18
Table S7	S19
Table S8	S19
Table S9	S20
Table S10	S20

Experimental S1. Characterization techniques

The morphology and hydrogels cross-section microstructure of DUT-67 samples were studied by SEM. Hydrogel samples were swelled in distilled water, lyophilized, and then cryofractured after a rapid freezing in liquid nitrogen. For image recording, hydrogel samples were coated with gold particles. PXRD patterns of DUT-67 samples were obtained in a Panalytical X´pert CuKα diffractometer operating at 2θ range = 5–70°, step size = 0.015°, exposure time = 10 s per step, at room temperature. Panalytical X´pert is a polycrystalline sample diffractometer with a Bragg Brentano geometry, a programmable slit, secondary graphite monochromator adjusted to copper radiation and a fast solid state PixCel detector adjusted to a 3.347° active length in 2θ(°). The equipment allows performing quality measurements for the subsequent data processing at the level of full profile adjustments without/with a structural model. ATR-FTIR, and FT-Raman spectra were recorded on a Nicolet iS50 spectrometer (Thermo Scientific) using a one-reflection diamond crystal. ATR-FTIR spectra were recorded with 32 scans at a resolution of 4 cm-1. FT-Raman spectra were acquired with an excitation laser beam of 1064 nm, 0.5 W power, resolution of 4 cm^{-1}, and 50 scans. All samples were previously oven-dried at 60°C for 24 h.

HRMAS NMR and ss-NMR spectra were acquired with a Bruker Avance-III HD spectrometer equipped with a 14.1 T narrow bore magnet. operating at Larmor frequencies of 600.09 and 150.91 MHz for ^{1}H and ^{13}C, respectively. Composite hydrogels were studied by ^{1}H NMR HRMAS by packing the D_2O-swelled sample into a 4 mm ZrO_2 HRMAS rotor with a 50 □L spherical insert. The sample was spun at a MAS rate of 4 kHz. A pre-saturation pulse (zgpr) was used for water-suppression in the ^{1}H experiments. Powdered samples were packed into 3.2 and 2.5 mm ZrO_2 rotors and rotated at room temperature at MAS rates of 15 or 32 kHz, respectively. ^{13}C CP-MAS experiments were done in a 3.2 mm MAS probe. Glycine was used as external reference compound for the recording of the ^{13}C spectra and to set the Hartmann−Hahn matching

condition in the CP-MAS experiments in ^{13}C spectra.[55] The contact time during CP was 2 ms. The SPINAL64 sequence (small phase incremental alternation with 64 steps)[56] was used for heteronuclear decoupling during acquisition. The 2D $^{1}H-^{13}C$ HETCOR experiment with frequency–switched Lee-Goldberg irradiation during the dipolar proton evolution in the solid state was recorded using a contact time of 2 ms.[57] ^{1}H-MAS and 2D homonuclear correlation experiments were recorded in a 2.5 mm MAS probe. The 2D ^{1}H-^{1}H SQ/DQ experiment was acquired with the back-to-back (BaBa) pulse sequence with excitation and reconversion times of two rotor period.[58] Chemical shifts for ^{13}C and ^{1}H (expressed in ppm) are relative to glycine and $(CH_3)_4Si$, respectively.

The TGA was recorded in a TGA-50 Shimadzu. Samples were previously oven-dried at 60°C for 24 h. The analysis was performed from 25 to 800 °C in a nitrogen atmosphere with a heating rate of 10°C min^{-1}.

XPS analysis was used to obtain quantitative and chemical state information on the surface of the materials, and was carried out with a Physical Electronics (Versa-Pro II) operating with a monochromatic X-ray source Al (k-alpha) of photons at 1486 eV under ultra-high vacuum using a pressure of 10^{-6} Pa. The XPS experimental results were analyzed using a 0.651 eV Au $4f_{7/2}$ line of full width at half maximum.

CO_2 high pressure adsorption isotherms were acquired in an ISorb-1 equipment from 0 to 30 bars, and after activating the sample at 120°C for 4 hours. The BET surface areas were calculated following the protocol described by Kim et al.[53] and considering the fitting of the data between 0.05 and 0.35 bars.

The swelling behavior of composite hydrogels was studied by the gravimetric method carried out in distilled water at 25°C in triplicate. In each experiment, the oven-dried hydrogel was immersed in water for 24 h. The swelled sample was then separated from

the solution and weighed after previous surface water removal with filter paper. Finally, the swelled samples were oven-dried at 60°C until constant weight was obtained. The swelling capacity (S) was calculated using the following equation:

$$S(\%) = \frac{M_s - M_d}{M_d} \times 100 \qquad (1)[59]$$

where M_s and M_d are the swelled and dried sample weight, respectively.

The pH at the point of zero charge (pH_{pzc}) of composite hydrogels was determined by the pH drift method.[60] The pH of NaCl solutions (10 mM) was adjusted over a range of 4 – 8 by adding either 0.1 M HCl or 0.1 M NaOH. Then, 50 mg of the swelled samples were immersed into 10 mL of each solution, incubated for 24 h at room temperature, and the final pH was measured. The pH value at which the curve of the final pH crosses the pHinitial=pHfinal lines is the pH_{pzc}.

The viscoelastic behavior was determined with an Anton Paar rotational rheometer (MCR-301). Parallel plates (d=25 mm) were used for the frequency sweep test from 0.1 to 500 s^{-1} using a strain value of 1% at 25°C. Measures fell within the lineal viscoelastic range, as assessed previously.

Experimental S2. Kinetic and Isotherm models

Adsorption kinetics

Pseudo-1st order and pseudo-2nd order were used as described by:[61],[62]

$$q_t = q_{eq}(1 - e^{-k_1.t}) \tag{1}$$

$$q_t = \frac{q_{eq}^2.k_2.t}{1+(q_{eq}.k_2.t)} \tag{2}$$

where q_t and q_{eq} are adsorption capacities at time t (h) and at equilibrium respectively (mg/g), k_1 (h^{-1}) and k_2 (g/mg h) are the sorption rate constants for the pseudo-1st and pseudo-2nd order models, respectively. The initial sorption rate h_0 (mg/g h) for the pseudo-2nd order kinetic model was written as: $h_0 = q_{eq}^2\ k_2$.

Considering $q_t = q_t$ at $t = t$ and $q_t = 0$ at $t = 0$, the Elovich rate equation becomes:[63]

$$q_t = \frac{1}{\beta}\ln\left(1 + (\alpha\cdot\beta\cdot t)\right) \tag{3}$$

where constant α (mg/g h) is the initial adsorption rate and β (g/mg) is related to the extent of surface coverage and the activation energy involved in chemisorption processes.[64] This equation assumes that the active sites of the sorbent are heterogeneous in nature and therefore exhibit different activation energies for chemisorption.[65]

The modified Freundlich model was originally developed by Kuo and Lotse and is described by:[66]

$$q_t = k_F.C_0.t^{1/m} \tag{4}$$

where k_F (L/g h) is the apparent adsorption rate constant, C_0 (mg/L) the initial sorbate concentration and m (dimensionless) is the Kuo-Lotse constant.[66] This model can

describe surface diffusion-controlled processes. Particularly, it can describe kinetics controlled by intra-particle diffusion when *m* approaches a value of 2.[62]

Adsorption isotherms

Adsorption capacities (q_{eq}) are expressed as the moles of sorbate per mass unit of sorbent (mmol/g) and determined as follows:

$$q_{eq} = (C_0 - C_{eq})V / m \quad (5)$$

where C_0 and C_{eq} are the initial and the equilibrium sorbate concentrations of the incubation solution respectively (mg/L), *V* is volume of solution (L) and *m* is the sorbate mass (g).

Two parameters adsorption isotherms

Langmuir and Freundlich models have been widely applied to the adjustment of biosorption equilibrium data. The former assumes that a sorbate interacts homogeneously, at homogeneous sorption sites, until a monolayer is formed in the sorbent surface. On the other hand, Freundlich model have proved to describe better the adjustment of sorbents with heterogeneous adsorption sites and dissimilar interactions.[61] Langmuir and Freundlich adsorption isotherms can be expressed using equations (7) and (8) respectively:[66]

$$q_{eq} = \frac{q_m.K_a.C_{eq}}{1 + K_a.C_{eq}} \quad (7)$$

$$q_{eq} = k.C_{eq}^{n} \quad (8)$$

where K_a is the adsorption equilibrium constant (L/mg), q_m is the maximum adsorption capacity (mg/g) and *k* and *n* are arbitrary parameters. The dimension of *k* depends on the value of *n*.

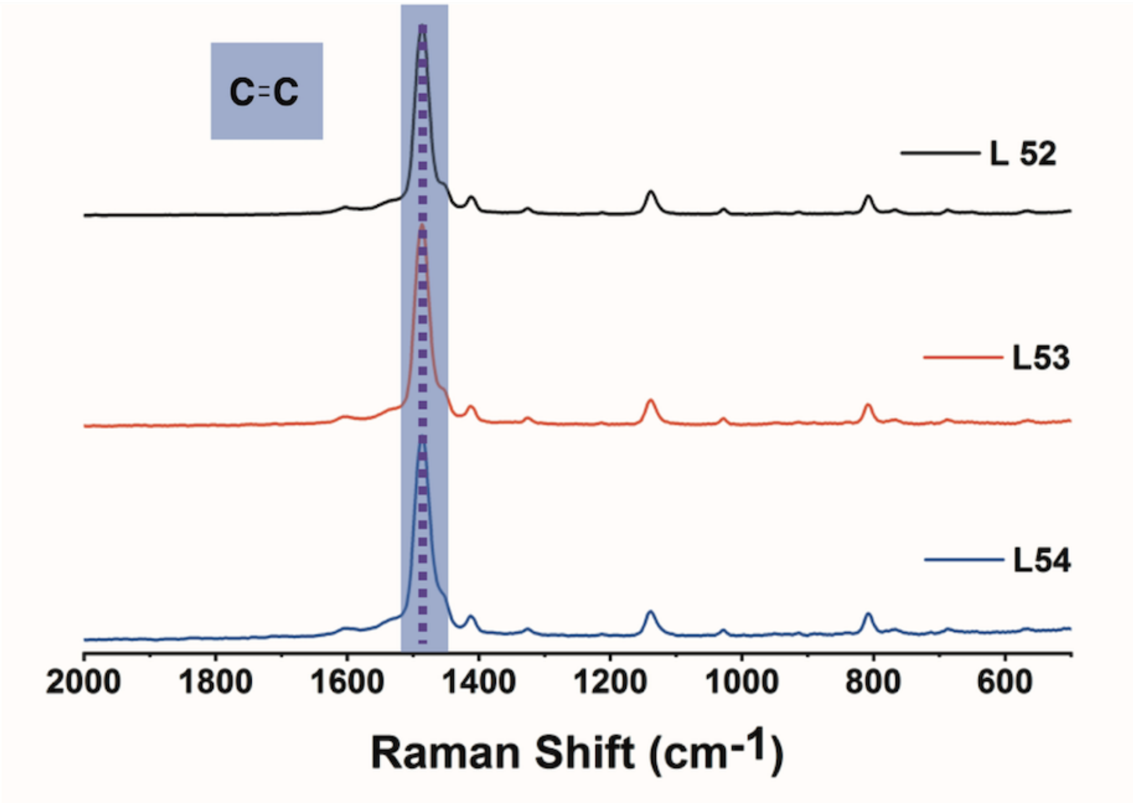

Figure S1. FT-Raman spectra f L52, L53, and L54.

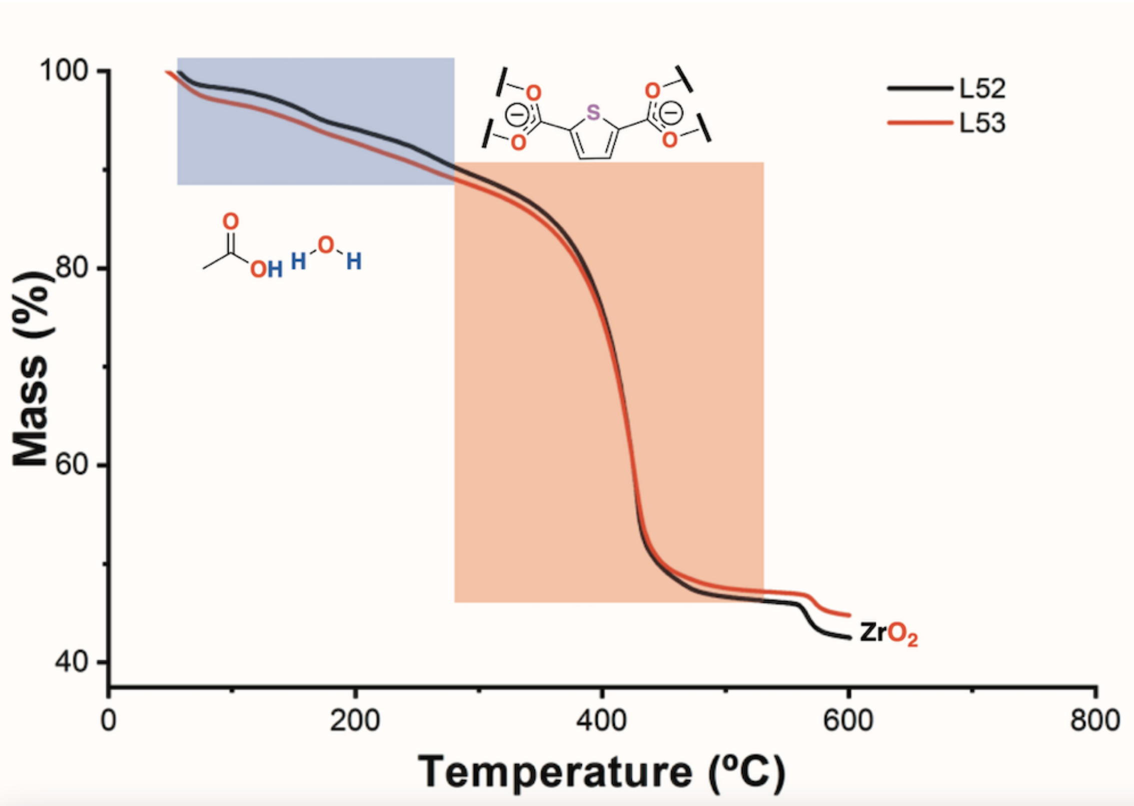

Figure S2. TGA curves of L52 and L53.

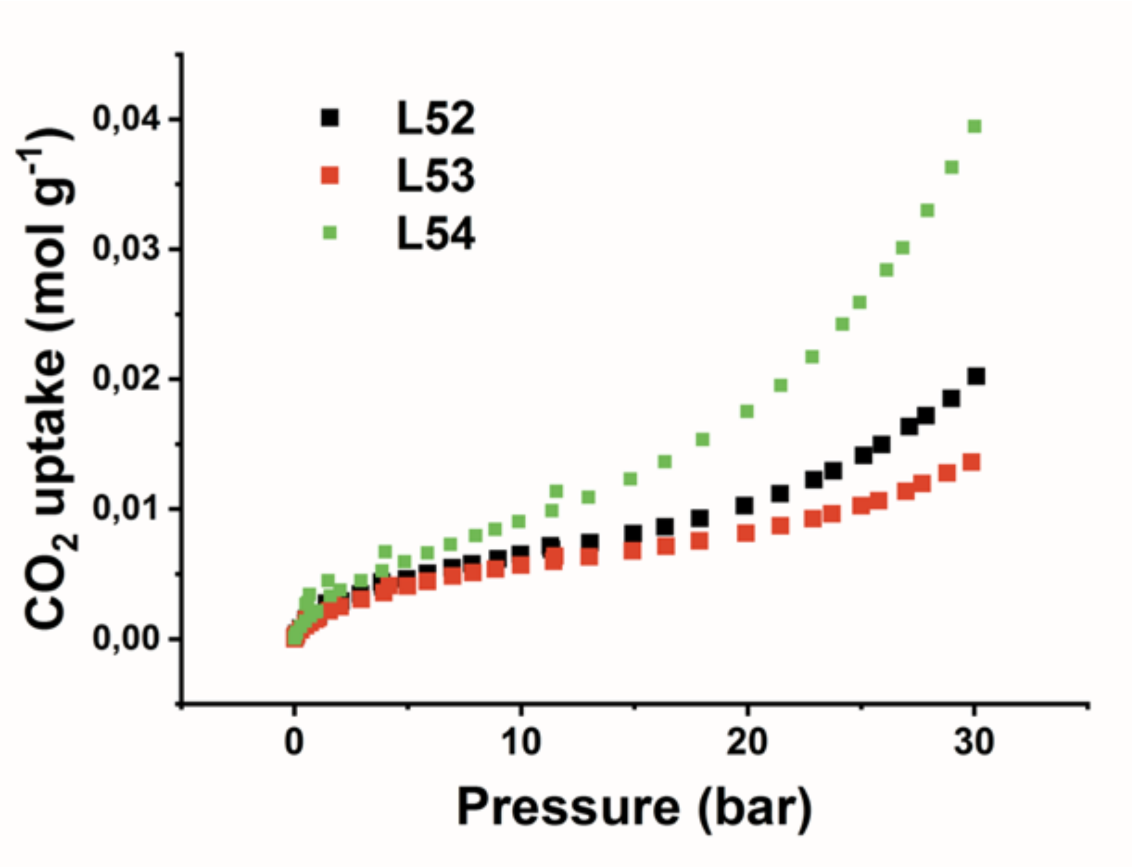

Figure S3. High pressure CO_2 adsorption isotherms at 0 ºC of L52, L53, and L54.

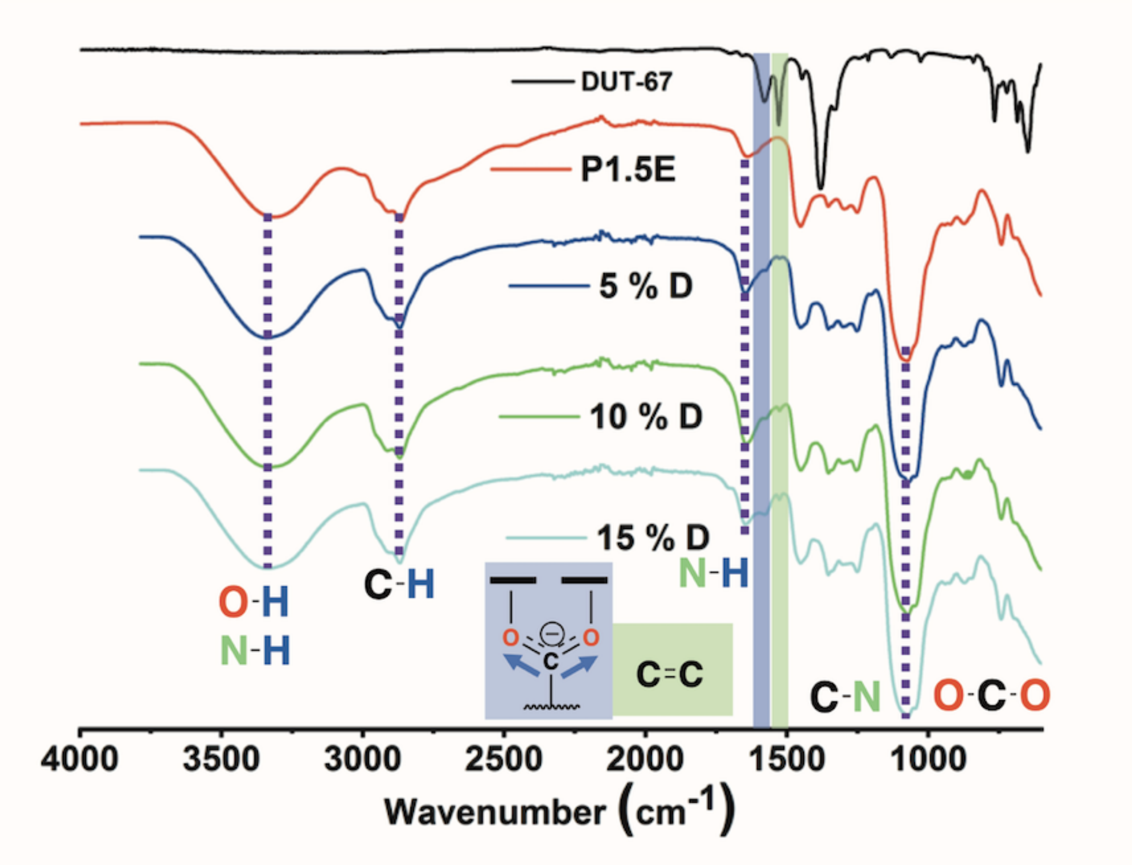

Figure S4. ATR-FTIR spectra of L54, P1.5E, 5%D, 10%D, and 15%D.

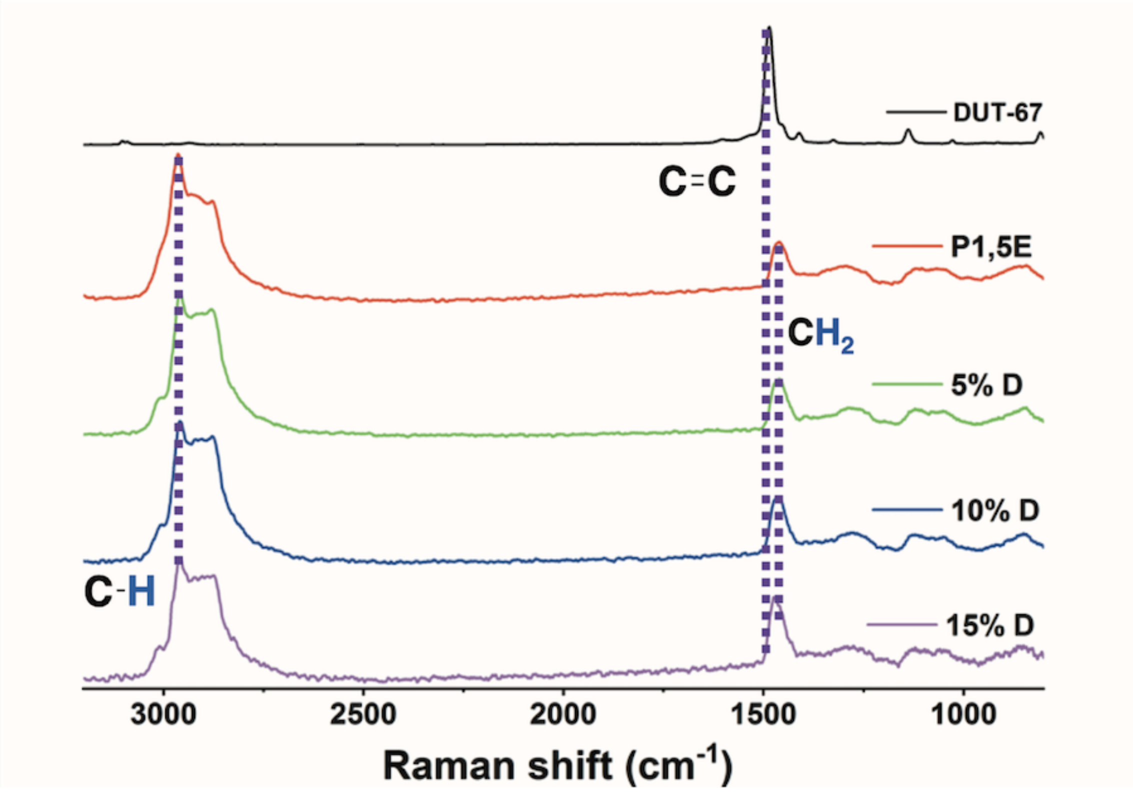

Figure S5. FT-RAMAN spectra of L54, P1.5E, 5%D, 10%D, and 15%D.

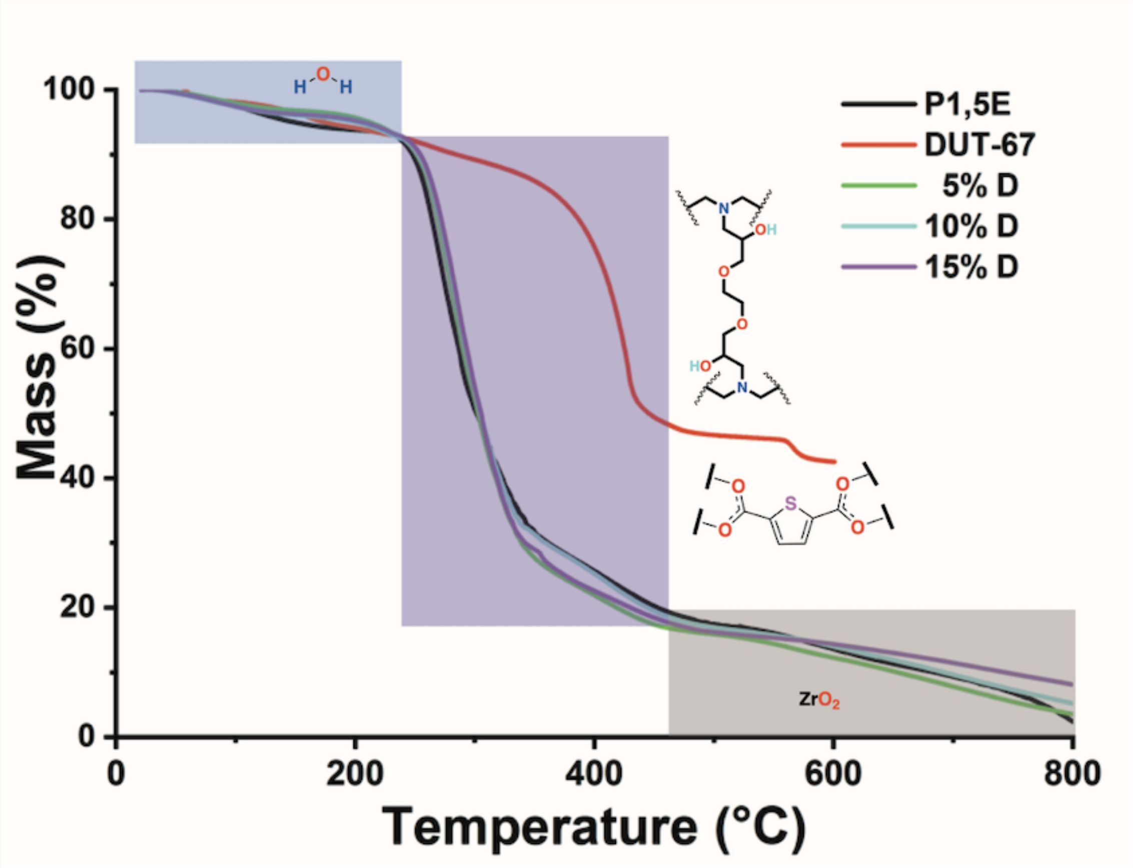

Figure S6. TGA curves of L54, P1.5E, 5%D, 10%D, and 15%D.

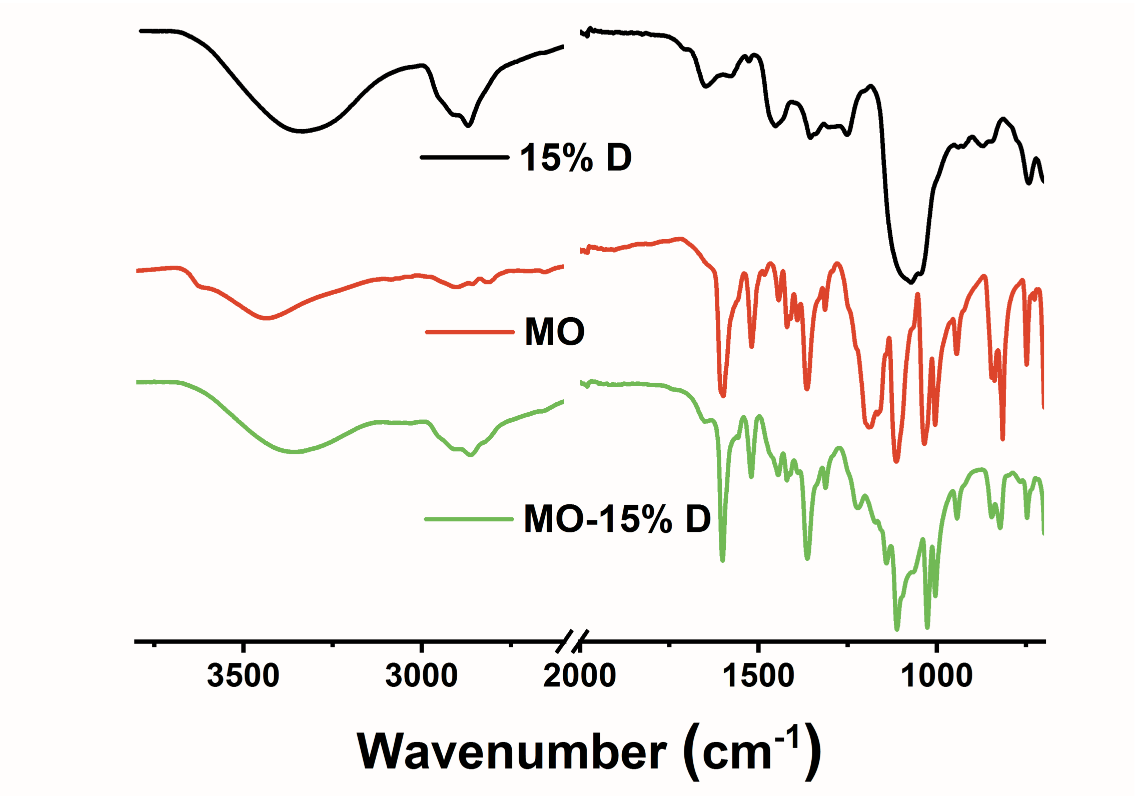

Figure S7. ATR-FTIR spectra of 15% D after MO adsorption.

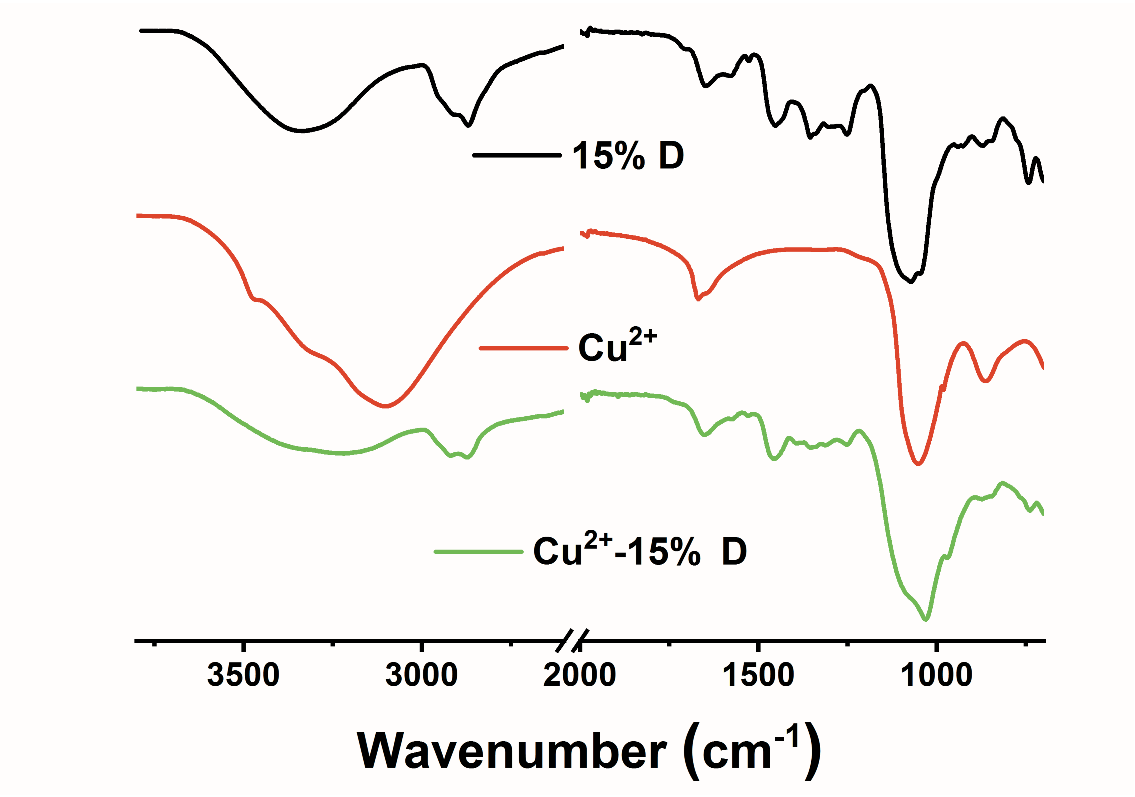

Figure S8. ATR-FTIR spectra of 15% D after Cu^{2+} adsorption.

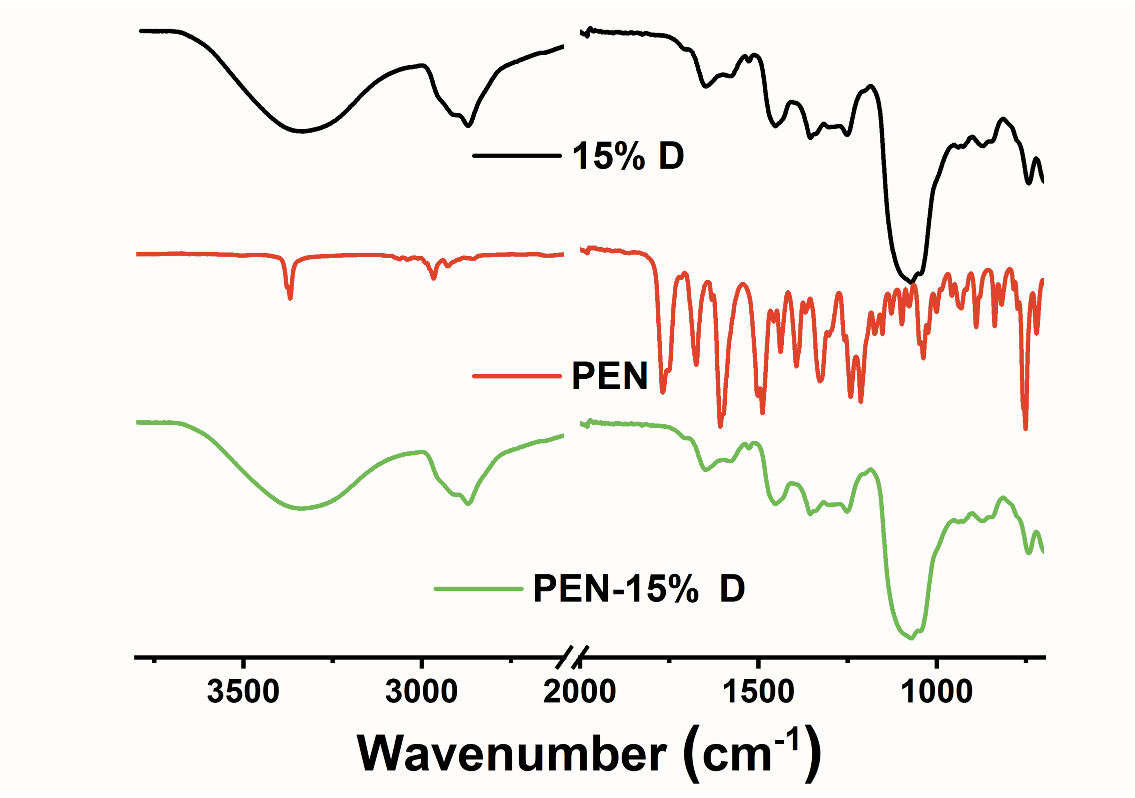

Figure S9. ATR-FTIR spectra of 15% D after PEN adsorption.

Table S1. Solvent (H_2O): modulator (CH_3CO_2H) proportions for DUT-67 synthesis

Sample	$ZrOCl_2.8H_2O$ (g)	H_2O (mL)	CH_3CO_2H (mL)	H_2TDC (g)
L52	6.440	50	50	2.290
L53	6.440	75	25	2.290
L54	6.440	95	5	2.290

Table S2. Fitting parameters from XPS data of L54

C 1s								
**Band**	**Pos**	**PosSep**	**B_FWHM**	**FWHM**	**Height**	**%Gauss**	**Area**	**%Area**
1	284.7	0	1.36	1.36	1540	89	2350	75.2
2	286.0	1.27	1.66	1.66	335	70	677	21.7
3	288.6	3.89	1.4	1.4	65	100	98	3.1
**O 1s**								
**Band**	**Pos**	**PosSep**	**B_FWHM**	**FWHM**	**Height**	**%Gauss**	**Area**	**%Area**
1	530.1	0	1.74	1.74	2223	96	4190	28.4
2	531.6	1.54	1.71	1.71	4991	100	9073	61.5
3	532.7	2.61	1.7	1.7	824	100	1491	10.1
**Zr 3d**								
**Band**	**Pos**	**PosSep**	**B_FWHM**	**FWHM**	**Height**	**%Gauss**	**Area**	**%Area**
1	182.5	0	1.7	1.7	4559	99	8315	59.9
2	185.0	2.43	1.7	1.7	3070	100	5571	40.1
**S 2p**								
**Band**	**Pos**	**PosSep**	**B_FWHM**	**FWHM**	**Height**	**%Gauss**	**Area**	**%Area**
1	164.1	0	1.67	1.67	452	80	879	66.7
2	165.3	1.18	1.67	1.67	241	94	439	33.3

Table S3 – Fitting parameters from XPS data of 15%D

			C 1s					
**Band**	**Pos**	**PosSep**	**B_FWHM**	**FWHM**	**Height**	**%Gauss**	**Area**	**%Area**
1	284.7	0	1.6	1.6	3091	95	5412	65.1
2	285.6	0.94	1.51	1.51	681	70	1251	15.0
3	288.8	4.09	1.4	1.4	1015	80	1656	19.9
			**O 1s**					
**Band**	**Pos**	**PosSep**	**B_FWHM**	**FWHM**	**Height**	**%Gauss**	**Area**	**%Area**
1	532.0	0	1.7	1.7	494	80	979	77.7
2	533.2	1.21	1.67	1.67	151	90	281	22.3
			**Zr 3d**					
**Band**	**Pos**	**PosSep**	**B_FWHM**	**FWHM**	**Height**	**%Gauss**	**Area**	**%Area**
1	182.5	0	1.59	1.59	12	80	22	59.9
2	184.9	2.43	1.59	1.59	8	80	15	40.1
			**N 1s**					
**Band**	**Pos**	**PosSep**	**B_FWHM**	**FWHM**	**Height**	**%Gauss**	**Area**	**%Area**
1	399.1	0	2	2	56	80	131	45.7
2	401.5	2.35	2.1	2.1	64	80	156	54.3

Table S4. Fitting parameters from XPS data of P1.5E

C 1s								
**Band**	**Pos**	**PosSep**	**B_FWHM**	**FWHM**	**Height**	**%Gauss**	**Area**	**%Area**
1	284.8	0	1.43	1.43	8273	92	13051	67.6
2	286.0	1.25	1.43	1.43	2361	100	3597	18.6
3	286.9	2.14	1.43	1.43	1209	100	1841	9.5
4	288.8	4	1.43	1.43	549	100	837	4.3
**O 1s**								
**Band**	**Pos**	**PosSep**	**B_FWHM**	**FWHM**	**Height**	**%Gauss**	**Area**	**%Area**
1	532.3	0	1.6	1.6	5288	90	9418	70
2	533.4	1.18	1.5	1.5	2311	80	4041	30
**N 1s**								
**Band**	**Pos**	**PosSep**	**B_FWHM**	**FWHM**	**Height**	**%Gauss**	**Area**	**%Area**
1	399.7	0	2	2	110	80	257	69.8
2	401.8	2.09	2	2	48	80	111	30.2

Table S5. Kinetic parameters for MO adsorption

		P1.5E		15% D	
**Model**	**Parameter**	**Value**	**Adjustment criteria**	**Value**	**Adjustment criteria**
Pseudo 1st order	q_e (mg L^{-1})	454±31	χ^2 = 0.7	480±14	χ^2 = 7.5
	k_1 (h^{-1})	0.94±0.14	RSS = 5.4	2.13±0.17	RSS = 60.02
Pseudo 2nd order	q_e (mg L^{-1})	608±53	χ^2= 0.5	553±13	χ^2= 2.3
	k_2 (g mg^{-1} h^{-1})	(13.5±3.8) x 10^{-4}	RSS = 4.3	(50±5.1) x 10^{-4}	RSS = 18.7
Elovich	α (mg g^{-1} h^{-1})	0.3±0.06	χ^2= 0.2	1.37±0.14	χ^2= 3.5
	β (g mg^{-1})	(32.4±0.9) x 10^{-4}	RSS = 1.9	(32.3±1.1) x 10^{-4}	RSS = 28.1
Modified Freundlich	k_F (L g^{-1} h^{-1})	4.4±0.1	χ^2 = 0.3	6.5±0.05	χ^2 = 1.2
	m	2.03±0.12	RSS = 2.1	3.9±0.2	RSS = 9.6

Table S6. Kinetic parameters for Cu^{2+} adsorption

		P1.5E		15% D	
**Model**	**Parameter**	**Value**	**Adjustment criteria**	**Value**	**Adjustment criteria**
Pseudo 1st order	q_e (mg L^{-1})	45.4±2.6	χ^2 = 0.6	49.8±0.5	χ^2 = 0.4
	k_1 (h^{-1})	0.99±0.14	RSS = 5.0	2.02±0.09	RSS = 4.0
Pseudo 2nd order	q_e (mg L^{-1})	58.9±5.7	χ^2= 0.7	56.3±0.8	χ^2= 0.4
	k_2 (g mg^{-1} h^{-1})	(15.2±5.0) x 10^{-3}	RSS = 6.2	(45.8±3.9) x 10^{-3}	RSS = 3.6
Elovich	α (mg g^{-1} h^{-1})	0.34±0.17	χ^2 = 1.1	1.3±0.3	χ^2 = 3.7
	β (g mg^{-1})	0.034±0.002	RSS = 9.6	0.003±0.002	RSS = 33.2
Modified Freundlich	k_F (L g^{-1} h^{-1})	1.9±0.1	χ^2 = 1.0	3.1±0.1	χ^2 = 2.1
	m	2.2±0.3	RSS = 9.4	3.9±0.4	RSS = 18.5

Table S7. Kinetic parameters for PEN adsorption

		P1.5E		15% D	
**Model**	**Parameter**	**Value**	**Adjustment criteria**	**Value**	**Adjustment criteria**
Pseudo 1st order	q_e (mg L^{-1})	67±4	χ^2 = 0.5	77±2	χ^2 = 0.4
	k_1 (h^{-1})	1.8±0.4	RSS = 4.2	3.8±0.7	RSS = 3.5
Pseudo 2nd order	q_e (mg L^{-1})	80±8	χ^2= 0.7	81±3	χ^2= 0.6
	k_2 (g mg^{-1} h^{-1})	(23.3±11.5) x 10^{-3}	RSS = 6.2	0.1±1.04	RSS = 5.3
Elovich	α (mg g^{-1} h^{-1})	0.69±0.49	χ^2 = 1.7	9.3±6.6	χ^2 = 1.0
	β (g mg^{-1})	0.023±0.003	RSS = 15.0	0.034±0.007	RSS = 9.2
Modified Freundlich	k_F (L g^{-1} h^{-1})	5.0±0.5	χ^2 = 1.3	6.1±0.2	χ^2 = 0.8
	m	3.2±1.0	RSS = 11.9	13.1±7.0	RSS = 7.7

Table S8. Isotherm model parameters for MO adsorption

		P1.5E		15%D	
**Model**	**Parameter**	**Value**	**Adjustment criteria**	**Value**	**Adjustment criteria**
Langmuir	q_m (mg g^{-1})	402±14	χ^2 = 790	473 ± 21	χ^2 = 1190
	k_a (L mg^{-1})	0.59±0.10	RSS = 11063	0.47 ± 0.08	RSS = 16656
Freundlich	k	176±14	χ^2= 1519	156 ± 13	χ^2= 1577
	n	0.25±0.03	RSS = 21268	0.25 ± 0.03	RSS = 31548

Table S9. Isotherm model parameters for Cu^{2+} adsorption

		P1.5E		15%D	
**Model**	**Parameter**	**Value**	**Adjustment criteria**	**Value**	**Adjustment criteria**
Langmuir	q_m (mg g^{-1})	74±6	χ^2 = 60	86 ± 6	χ^2 = 83
	k_a (L mg^{-1})	0.14±0.03	RSS = 1027	0.17 ± 0.04	RSS = 1417
Freundlich	k	15±3	χ^2= 108	21.7±4	χ^2= 135
	n	0.39±0.06	RSS = 1841	0.35±0.06	RSS = 2302

Table S10. Isotherm model parameters for PEN adsorption

		P1.5E		15%D	
**Model**	**Parameter**	**Value**	**Adjustment criteria**	**Value**	**Adjustment criteria**
Langmuir	q_m (mg $^{-1}$)	123±9	χ^2 = 129	127 ± 4	χ^2 = 132
	k_a (L mg^{-1})	0.30±0.07	RSS = 2712	0.96 ± 0.17	RSS = 3165
Freundlich	k	37±5	χ^2= 209	64±5	χ^2= 235
	n	0.38±0.05	RSS = 4387	0.25±0.03	RSS = 5634

References.

55. Hartmann, S.R.; Hahn, E.L. Nuclear Double Resonance in the Rotating Frame. *Physical Review* **1962**, *128*, 2042–2053, doi:10.1103/PhysRev.128.2042.

56. Fung, B.M.; Khitrin, A.K.; Ermolaev, K. An Improved Broadband Decoupling Sequence for Liquid Crystals and Solids. *Journal of Magnetic Resonance* **2000**, *142*, 97–101, doi:10.1006/jmre.1999.1896.

57. van Rossum, B.-J.; Förster, H.; de Groot, H.J.M. High-Field and High-Speed CP-MAS13C NMR Heteronuclear Dipolar-Correlation Spectroscopy of Solids with Frequency-Switched Lee–Goldburg Homonuclear Decoupling. *Journal of Magnetic Resonance* **1997**, *124*, 516–519, doi:10.1006/jmre.1996.1089.

58. Feike, M.; Demco, D.E.; Graf, R.; Gottwald, J.; Hafner, S.; Spiess, H.W. Broadband Multiple-Quantum NMR Spectroscopy. *J Magn Reson A* **1996**, *122*, 214–221, doi:10.1006/jmra.1996.0197.

59. Sun, H.; Zhan, J.; Chen, L.; Zhao, Y. Preparation of CTS/PAMAM/SA/Ca2+ Hydrogel and Its Adsorption Performance for Heavy Metal Ions. *Appl Surf Sci* **2023**, *607*, doi:10.1016/j.apsusc.2022.155135.

60. Lopez-Ramon, M.V.; Stoeckli, F.; Moreno-Castilla, C.; Carrasco-Marin, F. On the Characterization of Acidic and Basic Surface Sites on Carbons by Various Techniques. *Carbon N Y* **1999**, *37*, 1215–1221, doi:10.1016/S0008-6223(98)00317-0.

61. Ho, Y. The Kinetics of Sorption of Divalent Metal Ions onto Sphagnum Moss Peat. *Water Res* **2000**, *34*, 735–742, doi:10.1016/S0043-1354(99)00232-8.

62. Anirudhan, T.S.; Rejeena, S.R.; Tharun, A.R. Preparation, Characterization and Adsorption Behavior of Tannin-Modified Poly(Glycidylmethacrylate)-Grafted Zirconium Oxide-Densified Cellulose for the Selective Separation of Bovine Serum Albumin. *Colloids Surf B Biointerfaces* **2012**, *93*, 49–58, doi:10.1016/j.colsurfb.2011.12.010.

63. Cheung, C.W.; Porter, J.F.; Mckay, G. Sorption Kinetic Analysis for the Removal of Cadmium Ions from Effluents Using Bone Char. *Water Res* **2001**, *35*, 605–612.

64. Teng, H.; Hsieh, C.-T. Activation Energy for Oxygen Chemisorption on Carbon at Low Temperatures. *Ind Eng Chem Res* **1999**, *38*, 292–297, doi:10.1021/ie980107j.

65. Kuo, S.; Lotse, E.G. Kinetics of Phosphate Adsorption and Desorption by Hematite and Gibbsite. *Soil Sci* **1973**, *116*, 400–406, doi:10.1097/00010694-197312000-00002.

66. Suen, S.-Y. A Comparison of Isotherm and Kinetic Models for Binary-Solute Adsorption to Affinity Membranes. *Journal of Chemical Technology & Biotechnology* **1996**, *65*, 249–257, doi:10.1002/(SICI)1097-4660(199603)65:3<249::AID-JCTB411>3.0.CO;2-M.
